# Supplementary material for: Effectiveness of thoracic spine manipulation for upper quadrant musculoskeletal disorders: protocol for a systematic review
Source: BMJ Open. 2023 Sep 15;13(9):e076143. doi: 10.1136/bmjopen-2023-076143 (PMC10510929; doi:10.1136/bmjopen-2023-076143)
Supplement: Supplementary data [file bmjopen-2023-076143supp001.pdf]

PIO: What is the effectiveness of a thoracic high velocity low amplitude thrust (HVLAT) manipulation in patients with musculoskeletal disorders in the upper quadrant, a systematic review.

OVID search of Embase and Medline

Musculoskeletal adj2 (pain OR disorder* OR disease* OR symptom* OR problem*) OR Neck adj2 (pain OR disorder* OR disease* OR symptom* OR problem*) OR Headache adj2 (disorder* OR tension type OR cervicogenic OR migraine) OR (Cervical radiculopathy) OR (radicular adj2 (syndrome* OR pain)) OR Cervical adj2 (nerve root OR disc OR (disc disease) OR impingement) OR Shoulder adj2 (pain OR disorder* OR disease* OR symptom* OR problem* OR imping*) OR (adhesive capsulitis) OR (frozen shoulder) OR Elbow adj2 (pain OR disorder* OR disease* OR symptom* OR problem* OR tennis) OR epicondylalgia OR epicondylitis OR Temporomandibular adj2 (joint OR pain OR disorder* OR disease* OR symptom* OR problem*) OR Upper extremity adj2 (joint OR pain OR disorder* OR disease* OR symptom* OR problem*) OR Upper quadrant adj2 (joint OR pain OR disorder* OR disease* OR symptom* OR problem*)

AND

Thoracic OR (Thoracic adj2 spine) OR Thora*

AND

Spinal manipulation OR Manipulation OR manipulat* OR adjustment OR Chiroprac* OR (high adj2 (velocity OR low OR amplitude OR thrust)) OR treat* OR therapy OR intervention OR SMT OR thrust OR mobilis* OR mobiliz* OR mobilization OR mobilization OR manual therapy OR osteopat* OR physical therapy OR physio* OR rehab* OR management OR HVLA*

AND

Pain OR (numerical rating scale) OR NRS OR (numerical pain rating scale) OR NPRS OR Disability OR disable* OR (return to work) OR effectiveness OR effect* OR patient adj2 (reported OR outcome OR measur*) OR (recovery of function) OR activit* adj2 (daily OR living OR life) OR Physical adj2 (examination OR test) OR Range adj2 motion OR Muscle adj2 strength OR Psychosocial* OR (Neck disability index) OR NDI OR (Bournemouth adj2 neck) OR (patient specific functional scale) OR PSFS OR (shoulder pain and disability index) OR SPADI
AND

Web of Science:

Musculoskeletal NEAR/1 (pain OR disorder* OR disease* OR symptom* OR problem*) OR Neck NEAR/1 (pain OR disorder* OR disease* OR symptom* OR problem*) OR Headache NEAR/1 (disorder* OR “tension type” OR cervicogenic OR migraine) OR (Cervical radiculopathy) OR (radicular NEAR/1 (syndrome* OR pain)) OR Cervical NEAR/1 ((“nerve root” OR disc OR “disc disease”) OR impingement) OR Shoulder NEAR/1 (pain OR disorder* OR disease* OR symptom* OR problem* OR imping*) OR “adhesive capsulitis” OR “frozen shoulder” OR Elbow NEAR/1 (pain OR disorder* OR disease* OR symptom* OR problem* OR tennis) OR epicondylalgia OR epicondylitis OR Temporomandibular NEAR/1 (joint OR pain OR disorder* OR disease* OR symptom* OR problem*) OR Upper extremity NEAR/1 (joint OR pain OR disorder* OR disease* OR symptom* OR problem*) OR Upper quadrant NEAR/2 (joint OR pain OR disorder* OR disease* OR symptom* OR problem*)

AND

Thoracic OR (Thoracic NEAR/2 spine OR spinal) OR Thoracal

AND

Spinal manipulation OR Manipulation OR manipulat* OR adjustment OR Chiroprac* OR (high NEAR/1 (velocity OR low OR amplitude OR thrust)) OR treat* OR therapy OR intervention OR smt OR thrust OR mobilis* OR mobiliz* OR mobilization OR mobilization OR manual therapy OR osteopat* OR physical therapy OR physio* OR rehab* OR management OR HVLA*

AND

Pain OR measure* OR (numerical rating scale) OR NRS OR (numerical pain rating scale) OR NPRS OR Disability OR disable* OR (return to work) OR patient NEAR/1 (reported OR outcome OR measur*) OR (recovery of function) OR activit* NEAR/1 (daily OR living OR life) OR Physical NEAR/1 (examination OR test) OR Range NEAR/1 motion OR Muscle NEAR/1 strength OR Psychosocial* OR (Neck disability index) OR NDI OR (Bournemouth NEAR/1 neck) OR (patient specific functional scale) OR PSFS OR (shoulder pain and disability index) OR SPADI

AND

randomised OR randomized OR random* OR (Clinical trial) OR RCT

SCOPUS; also used for CINAHL

Musculoskeletal W/2 pain OR Musculoskeletal W/2 disorder* OR Musculoskeletal W/2 disease* OR Musculoskeletal W/2 symptom* OR Musculoskeletal W/2 problem* OR Neck W/2 pain OR Neck W/2 disorder* OR Neck W/2 disease* OR Neck W/2 symptom* OR Neck W/2 problem* OR Headache W/2 disorder* OR Headache W/2 “tension type” OR Headache W/2 cervicogenic OR Headache W/2 migraine OR “Cervical radiculopathy” OR radicular W/2 syndrome* OR radicular W/2 pain OR radicular W/2 Cervical W/2 “nerve root” OR Cervical W/2 disc OR Cervical W/2 “disc disease” OR Cervical W/2 impingement OR Shoulder W/2 pain OR Shoulder W/2 disorder* OR Shoulder W/2 disease* OR Shoulder W/2 symptom* OR Shoulder W/2 problem* OR Shoulder W/2 imping* OR “adhesive capsulitis” OR “frozen shoulder” OR Elbow W/2

pain OR Elbow W/2 disorder* OR Elbow W/2 disease* OR Elbow W/2 symptom* OR Elbow W/2 problem* OR Elbow W/2 tennis) OR epicondylalgia OR epicondylitis OR Temporomandibular W/2 joint OR Temporomandibular W/2 pain OR Temporomandibular W/2 disorder* OR Temporomandibular W/2 disease* OR Temporomandibular W/2 symptom* OR Temporomandibular W/2 problem* OR "Upper extremity" W/2 joint OR "Upper extremity" pain OR "Upper extremity" disorder* OR "Upper extremity" disease* OR "Upper extremity" symptom* OR "Upper extremity" problem* OR "Upper quadrant" W/2 joint OR "Upper quadrant" W/2 pain OR "Upper quadrant" W/2 disorder* OR "Upper quadrant" W/2 disease* OR "Upper quadrant" W/2 symptom* OR "Upper quadrant" W/2 problem*

AND

Thoracic OR Thoracic W/2 spine OR Thoracic W/2 spinal OR Thora*

AND

"Spinal manipulation" OR Manipulation OR manipulat* OR adjustment OR Chiroprac* OR high W/2 velocity OR high W/2 low OR high W/2 amplitude OR high W/2 thrust OR treat* OR therapy OR intervention OR SMT OR "spinal manipul* therapy" OR thrust OR mobilis* OR mobiliz* OR mobilization OR mobilization OR manual therapy OR osteopat* OR "physical therapy" OR physio* OR rehab* OR management OR HVLA* OR "manipulative technique*"

AND

Pain OR measure* OR "numerical rating scale" OR NRS OR "numerical pain rating scale" OR NPRS OR Disability OR disable* OR "return to work" OR effectiveness OR effect* OR patient W/4 reported OR patient W/4 outcome OR patient W/4 measur* OR "recovery of function" OR activit* W/2 daily OR activit* W/2 living OR activit* W/2 life OR Physical W/2 examination OR Physical W/2 test OR Range W/2 motion OR Muscle W/2 strength OR Psychosocial* OR "neck disability index" OR NDI OR Bournemouth W/2 neck OR Bournemouth W/2 question* OR "patient specific functional scale" OR PSFS OR "shoulder pain and disability index" OR spadi

AND

randomised OR randomized OR random* OR "Clinical trial" OR RCT

Index to Chiropractic Literature

'Thoracic manipulation' AND random*

PEDro database

Thorac* AND manip*
