# Supplementary material for: Effectiveness of thoracic spine manipulation for upper quadrant musculoskeletal disorders: protocol for a systematic review
Source: BMJ Open. 2023 Sep 15;13(9):e076143. doi: 10.1136/bmjopen-2023-076143 (PMC10510929; doi:10.1136/bmjopen-2023-076143)
Supplement: Author's manuscript [file bmjopen-2023-076143.draft_revisions.pdf]

BMJ Open is committed to open peer review. As part of this commitment we make the peer review history of every article we publish publicly available.

When an article is published we post the peer reviewers’ comments and the authors’ responses online. We also post the versions of the paper that were used during peer review. These are the versions that the peer review comments apply to.

The versions of the paper that follow are the versions that were submitted during the peer review process. They are not the versions of record or the final published versions. They should not be cited or distributed as the published version of this manuscript.

BMJ Open is an open access journal and the full, final, typeset and author-corrected version of record of the manuscript is available on our site with no access controls, subscription charges or pay-per-view fees (http://bmjopen.bmj.com).

If you have any questions on BMJ Open’s open peer review process please email info.bmjopen@bmj.com

Effectiveness of thoracic spine manipulation on upper quadrant musculoskeletal disorders; protocol for a systematic review.

Journal:	*BMJ Open*
Manuscript ID	bmjopen-2023-076143
Article Type:	Protocol
Date Submitted by the Author:	29-May-2023

Complete List of Authors:	Thoomes, Erik; University of Birmingham College of Life and Environmental Sciences, School of Sport, Exercise and Rehabilitation Sciences; Fysio-Experts, Research department<br>Tilborghs, Gus; Breederode College, Department of Manual Therapy<br>Heneghan, Nicola; University of Birmingham, School of Sport, Exercise and Rehabilitation Sciences<br>Falla, Deborah; University of Birmingham College of Life and Environmental Sciences, School of Sport, Exercise and Rehabilitation Sciences<br>de Graaf, Marloes; Breederode College, Department of Manual Therapy; Fysio-Experts, Research department

Keywords:	Systematic Review, Spine < ORTHOPAEDIC & TRAUMA SURGERY, Physical Therapy Modalities

SCHOLARONE™ Manuscripts

Effectiveness of thoracic spine manipulation on upper quadrant musculoskeletal disorders; protocol for a systematic review.

Erik Thoomes [1, 2], Gus Tilborghs [3], Nicola R Heneghan [1], Deborah Falla [1], Marloes de Graaf [2,3]

[1]Centre of Precision Rehabilitation for Spinal Pain (CPR Spine), School of Sport, Exercise and Rehabilitation Sciences, College of Life and Environmental Sciences, University of Birmingham, Birmingham. United Kingdom
[2] Fysio-Experts, Research department, Hazerswoude, The Netherlands
[3] Department of Manual Therapy, Breederode University of Applied Science, Rotterdam, the Netherlands

Word count:
Abstract: 249
Manuscript: 2034 (including in text references)

Contrubutor's email addresses and ORCID iDs
Erik Thoomes: erikthoomes@gmail.com 0000- 0001- 6375- 2267
Marloes de Graaf: marloesdegraaf@icloud.com 0000- 0002- 2865- 5389
Nicola Heneghan: n.heneghan@bham.ac.uk 0000-0001-7599-3674
Deborah Falla: d.falla@bham.ac.uk 0000- 0003- 1689- 6190
Gus Tilborghs: gusdevlieger@msn.com n/a

For peer review only

ABSTRACT

Introduction

Upper quadrant musculoskeletal disorders (UQMD), comprising of cranial, cervical, shoulder and upper extremity complaints, are among the most frequently reported disorders in clinical practice. Thoracic high velocity low amplitude thrust (Tx-HVLAT) manipulation is a form of conservative management recommended in systematic reviews as an effective treatment option for aspects of UQMD disorders such headache, shoulder pain and lateral elbow pain. However, no recent systematic reviews have assessed the effectiveness across UQMD. Therefore, this systematic review aims to update the current evidence on the effectiveness of Tx-HVLAT for patients with UQMD on 1) patient reported outcomes, 2) performance measures or 3) psychosocial outcomes.

Methods and analysis

Following guidelines as advised by the Cochrane Back Review Group and compliant with PRISMA-P guidelines, published randomised controlled trials will be included Two review authors will independently assess the risk of bias using the Cochrane Back Review Group's recommended ROB2 tool and will independently extract the data using a standardized data extraction form. Overall quality of the evidence will be evaluated using the GRADE method.

For continuous data, we will calculate standardized mean differences with 95% confidence intervals (SMD; 95%CI). For dichotomous outcomes, Relative Risks (RRs) and 95%CIs will be calculated. Where possible we will present a subgroup analysis by disorder. For pooling a random-effects model will be used.

Ethics and dissemination

The study findings will be submitted to a relevant peer-reviewed journal for dissemination and then presented at relevant conferences.

Registration details

This systematic review was registered in the PROSPERO database (CRD42023429996).

KEYWORDS

Thoracic spine; spinal manipulation; high velocity low amplitude; effectiveness; systematic review

For peer review only

INTRODUCTION

Upper quadrant musculoskeletal disorders (UQMD), comprising of cranial, cervical, shoulder and upper extremity complaints, are among the most frequently reported disorders in clinical practice of both general physicians and physiotherapy (Walker-Bone et al. 2003, Huisstede et al. 2006). They are the second most common cause of work-related musculoskeletal disorders, with only lower back pain being more common (Executive 2016).

Most clinical guidelines for cervical and upper limb disorders suggest non-surgical management, such as physiotherapy, as a first treatment option for UQMD (Hegmann et al. 2013, Blanpied et al. 2017, Bier et al. 2018, Yu et al. 2021, Lafrance et al. 2022, Lapner et al. 2022). Spinal manipulation is one such management approach used in clinical practice. Manipulation is defined as "a passive, high velocity, low amplitude thrust (HVLAT) applied to a joint complex within its anatomical limit with the intent to restore optimal motion, function, and/or to reduce pain" in the International Federation of Orthopaedic Manipulative Physical Therapists' (IFOMPT) "Glossary of Terms" (Rusthon A 2012). Thoracic high velocity low amplitude thrust (Tx-HVLAT) manipulation is an intervention used by both orthopaedic manipulative physical therapists as well as chiropractors (Triano et al. 2003, Heneghan et al. 2020). In addition to the emerging evidence supporting its use for UQMD, the thoracic spine is also the most commonly manipulated spinal region (Adams and Sim 1998, Puentedura et al. 2017). One of the reasons for this has been linked to the theory of regional interdependence with the thoracic spine being viewed as a silent contributor to clinical presentations where a pain source lies elsewhere (Heneghan and Rushton 2016).

Tx-HVLAT is a recommended best practice management option for individuals with neck pain (Cross et al. 2011, Huisman et al. 2013, Côté et al. 2016, Blanpied et al. 2017, Masaracchio et al. 2019, Thoomes et al. 2022, Tsegay et al. 2023). Additionally, recent evidence from systematic reviews supports Tx-HVLAT also as an effective treatment option for other upper quadrant disorders such headache (Bini et al. 2022), shoulder pain (Strunce et al. 2009, Sanzo et al. 2016) and lateral elbow pain (Zunke et al. 2020). However, no recent systematic reviews have assessed the effectiveness across UQMD.

Therefore, this systematic review aims to update the current evidence on the effectiveness of Tx-HVLAT for patients with UQMD on 1) patient reported outcomes, 2) performance measures or 3) psychosocial outcomes.

METHODS

Protocol and Registration

This systematic review was registered in the PROSPERO database (CRD42023429996) and written in compliance with PRISMA-2020 guidelines (Page et al. 2021).

For peer review only

Selection Criteria

Inclusion criteria

Published randomised clinical trials (RCTs) for which full texts are available in English, Dutch, or German will be included.

Exclusion criteria

Abstracts for which full reports are not available or studies without the outcome of interest will be excluded.

Participants

Adult patients (age ≥ 18 years), with either short-term (less than 3 months), intermediate (3 months to 1 year) or long-term (more than 1 year) UQMD treated in primary care, hospitals or occupational settings will be included. For the purpose of this review, UQMD comprises of complaints of pain and/or disability in the head, neck, upper thoracic spine or upper extremity or complaints having been classified or labelled as UQMD .

Interventions

Studies using Tx-HVLAT (with or without cavitation) as an intervention for UQMD will be included. The technique could be provided once or multiple times to a single spinal region or various spinal regions during a single session or over multiple sessions. Co-interventions can also be included within the treatment session if these were also included in the comparison group. This allows for differences in treatment effect to be attributed to the addition of Tx-HVLAT in the experimental group.

Comparison

Comparisons which will be evaluated shall consist of: (a) placebo, waiting list control or no treatment or (b) other type(s) of conservative treatment.

Outcome measures

Following guidelines as advised by the Cochrane Back Review Group in establishing our primary outcomes, studies will be included that used at least one of the outcome measures that are considered to be the most important, namely: pain intensity, global perceived effect (e.g. proportion of patients recovered, subjective improvement of symptoms), disability (e.g. Neck Disability Index (NDI), Bournemouth Neck Questionnaire (NBQ), Shoulder Pain and Disability Index (SPADI)), return to work (e.g. days off work) or quality of life (e.g. EQ-5D).

Outcomes of physical examinations (e.g. range of motion, spinal flexibility, muscle strength, upper limb nerve tension testing), and psycho-social outcomes (e.g. anxiety, depression, pain behaviour) will be considered as secondary outcomes. Other outcomes such as drug consumption or adverse side effects will also be considered as secondary outcomes.

Search strategy

For peer review only

The search strategy will follow the recommendation by the Cochrane Handbook for Systematic Review of Interventions (Higgins et al. 2019). Electronic searches will include Cochrane Controlled Trials Register, MEDLINE, EMBASE, CINAHL and Index to Chiropractic Literature. We will use MeSH (Medline), Thesaurus (EMBASE, CINAHL) and free text words. Combinations will be made based on a) localisation (e.g. head, neck, thoracic spine or upper extremity); b) disorder (e.g. UQMD, headache, neck pain, thoracic spinal pain, shoulder pain, elbow pain or upper extremity disorders); c) intervention (e.g. HVLAT, manipulation, manual therapy, physiotherapy, physical therapy, chiropractic) and d) design: (randomised clinical trial or randomised controlled trial). Manual searches of published review bibliographies and reference lists of primary studies will be undertaken to search for possible studies not captured by the electronic searches (**see appendix 1**).

A research librarian together with a review author (ET) will perform the electronic searches. The search results will be uploaded and managed using EndNote v20 software (Clarivate Analytics, London UK) Two reviewer authors (ET,GT) will independently screen and select potentially eligible studies. Firstly, the title and abstract will be screened for eligibility. Secondly, the full text papers will be assessed to ascertain whether the study meets the inclusion criteria regarding design, participants, and interventions. Disagreements on inclusion will be resolved by discussion or through arbitration by a third review author (MdG).

Risk of bias assessment

Two review authors (ET,GT) will independently assess the risk of bias (ROB) using the Cochrane Back Review Group's recommended ROB2 tool (Sterne et al. 2019). This tool is structured into 5 different domains of bias (arising from the randomisation process, due to deviations from intended interventions, due to missing outcome data, in measurement of the outcome and in selection of the reported result). Within each domain, the assessment comprises: a series of signalling questions, a judgment about risk of bias for the domain, free text boxes to justify responses to the signalling questions and risk-of-bias judgments and optional free text boxes to predict (and explain) the likely direction of bias. When disagreement persists, a third review author (MdG) will be consulted. A low risk of bias is defined as being judged to be at low risk of bias for all domains for the result (Atkins et al. 2004).

Data extraction

Using a standardized data extraction form, two reviewer authors (ET,GT) will independently extract the data (including sample size, participant characteristics, in- & exclusion criteria, type of UQMD, types of interventions and comparators, outcome measures, follow up times and results) of the included RCTs. They will compare their forms for consistency, precision and accuracy. In cases of uncertainly about the data extracted, a third review author (MdG) will be consulted.

Data analysis

For peer review only

For continuous data, we will calculate standardized mean differences with 95% confidence intervals (SMD; 95%CI). SMD will be used because different measures are frequently used to address the same clinical outcome. Where applicable, the weighted mean difference (WMD) will be calculated. All data from Visual Analogue Scales (VAS) or Numerical Rating Scales (NRS) will be converted to scales ranging from 0-100, where necessary. For dichotomous outcomes, Relative Risks (RRs) and 95% Confidence Interval (CI) will be calculated. If the published article does not provide enough data, we will contact the original authors in an effort to retrieve additional necessary data, with a reminder being sent after two weeks.

Where possible we will present a subgroup analysis by disorder (e.g. neck pain, shoulder pain, headache). Prior to pooling, clinical heterogeneity sources such as differences in participant characteristics (e.g., age, baseline disease severity, ethnicity, comorbidities), types or timing of outcome measurements and intervention characteristics (e.g., dose, frequency of dose, training of interventionists) will be assessed through discussion with the research team (Higgins et al. 2022). For pooling a random-effects model will be used (Dettori et al. 2022).

RevMan Analyses (RevMan 5.3) will be used to analyse the data. The inter-observer reliability of the risk of bias assessments will be calculated using Kappa and categorised agreement as poor (0.0), slight (0.0-0.2), fair (0.21-0.4), moderate (0.41-0.6), substantial (0.61-0.8), or almost perfect (0.81-1.0) (Landis and Koch 1977).

For peer review only

Strength of the evidence

The overall quality of the evidence will be evaluated using the GRADE method (GRADE-Pro 2013) . The quality of the evidence will be based upon five principal factors: (1) limitations in study design (downgraded when >25% of the participants are from studies with a high risk of bias), (2) inconsistency of results [downgraded when there is statistical heterogeneity (I^2 >40%) or inconsistent findings (defined as ≤75% of the participants reporting findings in the same direction)], (3) indirectness (e.g. generalizability of the findings), (4) imprecision (downgraded when the total number of participants across studies is <300 for each outcome) and (5) other considerations, such as reporting bias. The quality of the evidence will be downgraded by one level when one of the factors described above is met (Atkins et al. 2004, Brozek et al. 2009).

Two independent reviewers (ET,GT) will grade the quality of evidence. Single studies will be considered inconsistent and imprecise (i.e. sparse data) and providing ‘‘low quality evidence’’, which can be further downgraded to ‘‘very low quality evidence’’ if there are also limitations in design or indirectness. The following grading of quality of the evidence will be applied (GRADE-Pro 2013):

- High quality evidence: further research is very unlikely to change confidence in estimate of effect.
- Moderate quality evidence: further research is likely to have an important impact on confidence in estimate of effect and may change the estimate.
- Low quality evidence: further research is very likely to have an important impact on confidence in estimate of effect and is likely to change the estimate.

- Very low quality evidence: very little confidence in the effect estimate.
- No evidence: no RCTs were identified that addressed this outcome

Dissemination plan

To ensure methodological rigour and ensure publication bias, this study protocol will be submitted to an open access peer-reviewed journal. The study findings will be submitted to a relevant peer-reviewed journal for dissemination and then presented at relevant conferences.

Patient and public involvement

The research question in this study forms part of a larger discussion within our patient and public involvement meetings as part of an existing programme of a multi-centre research programme that is focused on UQMD. Patients will not be involved in the analysis and data collection of the study.

Strengths and limitations of this study

- Ensuring methodological rigor by publishing the study protocol.
- This study follows Cochrane Handbook for Systematic Review of Interventions guidelines.
- Risk of bias (ROB) will be assessed using the Cochrane Back Review Group's recommended ROB2 tool.
- This study adheres to PRISMA-P guidelines.
- Strength of the evidence will be evaluated the by using the GRADEpro system.

DISCUSSION

Findings from this study may assist clinicians and researchers in formulating an individualised management plan for patients with UQMD. By assessing the effectiveness of Tx-HVLAT for different UQMD, clinicians will be better able to choose when to incorporate Tx-HVLAT as an effective treatment modality in evidence based multi-modal management strategies, instead of using a standardised 'one size fits all' approach. Findings from this study will also serve a need both clinically and within the contemporary literature to inform further research (e.g. on efficacy, cost effectiveness, etc.). We also aim to contrast this study's findings with previously published systematic reviews (Cross et al. 2011, Huisman et al. 2013, Sanzo et al. 2016, Masaracchio et al. 2019, Bini et al. 2022, Tsegay et al. 2023).

CONCLUSION

Thoracic spine manipuation (Tx-HVLAT) is one of the most often used treatment modality in multi-modal management strategies, not only for thoracic spinal pain but also for many other upper quadrant musculoskeletal disorders (UQMD). In order to collate, assess and update the current evidence of the effectiveness of Tx-HVLAT for

For peer review only

patients with UQMD, a systematic review has been designed. Findings from this study will facilitate the decision-making of clinicians in formulating individualised multi-modal management plans and the use of Tx-HVLAT through the natural course of recovery for patients with UQMD.

Twitter

Erik Thoomes @Fysio_Experts
Deborah Falla @Deb_Falla
Nicola Heneghan @HeneghanNicola

Author contributions

All authors devised the focus of this systematic review. ET and GT are PGR students, MdG is the lead supervisor, NH and DF are co-researchers. ET drafted the initial protocol manuscript with lead and co-supervisors providing guidance on methodological decisions and proposed analyses. All authors have contributed subject-specific expertise. All authors will contribute to data interpretation, conclusions and dissemination. All authors have read, contributed to and agreed to the final manuscript. DF and MdG are both guarantors of the study.

Funding

This study will be conducted as part of a Post Graduate Research project though the University of Birmingham, UK and as such received no specific grant from any funding agency in the public, commercial or not-for-profit sectors.

Competing interests

None declared.

Patient consent for publication

Not required.

Provenance and peer review

Not commissioned; externally peer reviewed.

Open access

This is an open access article distributed in accordance with the Creative Commons Attribution Non Commercial (CC BY-NC 4.0) license, which permits others to distribute, remix, adapt, build upon this work non-commercially, and license their derivative works on different terms, provided the original work is properly cited, appropriate credit is given, any changes made indicated, and the use is non-commercial.

Data statement section

Technical appendix, statistical code, and dataset available from the University of Birmingham eData Repository available at: https://edata.bham.ac.uk/

For peer review only

REFERENCES

Adams, G. and J. Sim (1998). "A survey of UK manual therapists' practice of and attitudes towards manipulation and its complications." Physiother Res Int **3**(3): 206-227.

Atkins, D., D. Best, P. A. Briss, M. Eccles, Y. Falck-Ytter, S. Flottorp, G. H. Guyatt, R. T. Harbour, M. C. Haugh, D. Henry, S. Hill, R. Jaeschke, G. Leng, A. Liberati, N. Magrini, J. Mason, P. Middleton, J. Mrukowicz, D. O'Connell, A. D. Oxman, B. Phillips, H. J. Schünemann, T. Edejer, H. Varonen, G. E. Vist, J. W. Williams, Jr. and S. Zaza (2004). "Grading quality of evidence and strength of recommendations." Bmj **328**(7454): 1490.

Bier, J. D., W. G. M. Scholten-Peeters, J. B. Staal, J. Pool, M. W. van Tulder, E. Beekman, J. Knoop, G. Meerhoff and A. P. Verhagen (2018). "Clinical Practice Guideline for Physical Therapy Assessment and Treatment in Patients With Nonspecific Neck Pain." Phys Ther **98**(3): 162-171.

Bini, P., D. Hohenschurz-Schmidt, V. Masullo, D. Pitt and J. Draper-Rodi (2022). "The effectiveness of manual and exercise therapy on headache intensity and frequency among patients with cervicogenic headache: a systematic review and meta-analysis." Chiropr Man Therap **30**(1): 49.

Blanpied, P. R., A. R. Gross, J. M. Elliott, L. L. Devaney, D. Clewley, D. M. Walton, C. Sparks and E. K. Robertson (2017). "Neck Pain: Revision 2017." J Orthop Sports Phys Ther **47**(7): A1-a83.

Brozek, J. L., E. A. Akl, P. Alonso-Coello, D. Lang, R. Jaeschke, J. W. Williams, B. Phillips, M. Lelgemann, A. Lethaby, J. Bousquet, G. H. Guyatt and H. J. Schünemann (2009). "Grading quality of evidence and strength of recommendations in clinical practice guidelines. Part 1 of 3. An overview of the GRADE approach and grading quality of evidence about interventions." Allergy **64**(5): 669-677.

Côté, P., J. J. Wong, D. Sutton, H. M. Shearer, S. Mior, K. Randhawa, A. Ameis, L. J. Carroll, M. Nordin, H. Yu, G. M. Lindsay, D. Southerst, S. Varatharajan, C. Jacobs, M. Stupar, A. Taylor-Vaisey, G. van der Velde, D. P. Gross, R. J. Brison, M. Paulden, C. Ammendolia, J. David Cassidy, P. Loisel, S. Marshall, R. N. Bohay, J. Stapleton, M. Lacerte, M. Krahn and R. Salhany (2016). "Management of neck pain and associated disorders: A clinical practice guideline from the Ontario Protocol for Traffic Injury Management (OPTIMa) Collaboration." European Spine Journal **25**(7): 2000-2022.

Cross, K. M., C. Kuenze, T. L. Grindstaff and J. Hertel (2011). "Thoracic spine thrust manipulation improves pain, range of motion, and self-reported function in patients with mechanical neck pain: a systematic review." J Orthop Sports Phys Ther **41**(9): 633-642.

Dettori, J. R., D. C. Norvell and J. R. Chapman (2022). "Fixed-Effect vs Random-Effects Models for Meta-Analysis: 3 Points to Consider." Global Spine J **12**(7): 1624-1626.

Executive, H. (2016). Work-related Musculoskeletal Disorders (WRMSDs) Statistics in Great Britain. 2017.

GRADE-Pro (2013). GRADE handbook for grading quality of evidence and strength of recommendations. Updated, The GRADE Working Group.

Hegmann, K. T., H. E. Hoffman, R. M. Belcourt, K. Byrne, L. Glass, J. M. Melhorn, J. Richman, P. Zinni, 3rd, M. S. Thiese, U. Ott, K. Tokita, D. G. Passey, A. C. Effiong, R. B. Robbins and J. A. Ording (2013). "ACOEM practice guidelines: elbow disorders." J Occup Environ Med **55**(11): 1365-1374.

Heneghan, N. R., E. J. Puentedura, I. Arranz and A. Rushton (2020). "Thoracic thrust joint manipulation: An international survey of current practice and knowledge in IFOMPT member countries." Musculoskelet Sci Pract **50**: 102251.

Heneghan, N. R. and A. Rushton (2016). "Understanding why the thoracic region is the 'Cinderella' region of the spine." Man Ther **21**: 274-276.

Higgins, J. P., J. Thomas, J. Chandler, M. Cumpston, T. Li, M. J. Page and V. A. Welch (2019). Cochrane handbook for systematic reviews of interventions, John Wiley & Sons.

Higgins, J. P., J. Thomas, J. Chandler, M. Cumpston, T. Li, M. J. Page and V. A. Welch (2022). Cochrane handbook for systematic reviews of interventions; v 6.3, John Wiley & Sons.

For peer review only

Huisman, P. A., C. M. Speksnijder and A. de Wijer (2013). "The effect of thoracic spine manipulation on pain and disability in patients with non-specific neck pain: a systematic review." Disabil Rehabil **35**(20): 1677-1685.
Huisstede, B. M., S. M. Bierma-Zeinstra, B. W. Koes and J. A. Verhaar (2006). "Incidence and prevalence of upper-extremity musculoskeletal disorders. A systematic appraisal of the literature." BMC Musculoskelet Disord **7**: 7.
Lafrance, S., M. Charron, J. S. Roy, J. O. Dyer, P. Frémont, C. E. Dionne, J. C. Macdermid, M. Tousignant, A. Rochette, P. Doiron-Cadrin, V. Lowry, N. Bureau, M. Lamontagne, E. Sandman, M. F. Coutu, P. Lavigne and F. Desmeules (2022). "Diagnosing, Managing, and Supporting Return to Work of Adults With Rotator Cuff Disorders: A Clinical Practice Guideline." J Orthop Sports Phys Ther **52**(10): 647-664.
Landis, J. R. and G. G. Koch (1977). "The measurement of observer agreement for categorical data." Biometrics **33**(1): 159-174.
Lapner, P., A. Alfonso, J. Herbert-Davies, J. W. Pollock, J. Marsh and G. King (2022). "Position statement: nonoperative management of lateral epicondylitis in adults." Can J Surg **65**(5): E625-e629.
Masaracchio, M., K. Kirker, R. States, W. J. Hanney, X. Liu and M. Kolber (2019). "Thoracic spine manipulation for the management of mechanical neck pain: A systematic review and meta-analysis." PLoS One **14**(2): e0211877.
Page, M. J., J. E. McKenzie, P. M. Bossuyt, I. Boutron, T. C. Hoffmann, C. D. Mulrow, L. Shamseer, J. M. Tetzlaff, E. A. Akl, S. E. Brennan, R. Chou, J. Glanville, J. M. Grimshaw, A. Hróbjartsson, M. M. Lalu, T. Li, E. W. Loder, E. Mayo-Wilson, S. McDonald, L. A. McGuinness, L. A. Stewart, J. Thomas, A. C. Tricco, V. A. Welch, P. Whiting and D. Moher (2021). "The PRISMA 2020 statement: an updated guideline for reporting systematic reviews." BMJ **372**: n71.
Puentedura, E. J., R. Slaughter, S. Reilly, E. Ventura and D. Young (2017). "Thrust joint manipulation utilization by U.S. physical therapists." J Man Manip Ther **25**(2): 74-82.
Rusthon A, e. a. (2012). "The Standards Document, Glossary of Terms." from http://www.ifompt.com/Standards/SC+Glossary.html.
Sanzo, P., E. Yeung, L. Levesque, E. Maheu, T. Woodard, S. Michels and L. Bouin (2016). "Is thoracic manipulation effective in managing shoulder dysfunction–A systematic review." Manual Therapy **100**(25): e154.
Sterne, J. A. C., J. Savović, M. J. Page, R. G. Elbers, N. S. Blencowe, I. Boutron, C. J. Cates, H. Y. Cheng, M. S. Corbett, S. M. Eldridge, J. R. Emberson, M. A. Hernán, S. Hopewell, A. Hróbjartsson, D. R. Junqueira, P. Jüni, J. J. Kirkham, T. Lasserson, T. Li, A. McAleenan, B. C. Reeves, S. Shepperd, I. Shrier, L. A. Stewart, K. Tilling, I. R. White, P. F. Whiting and J. P. T. Higgins (2019). "RoB 2: a revised tool for assessing risk of bias in randomised trials." Bmj **366**: l4898.
Strunce, J. B., M. J. Walker, R. E. Boyles and B. A. Young (2009). "The immediate effects of thoracic spine and rib manipulation on subjects with primary complaints of shoulder pain." J Man Manip Ther **17**(4): 230-236.
Thoomes, E., M. T. Graaf, J. A. Cleland, A. Gallina and D. Falla (2022). "Timing of Evidence-Based Nonsurgical Interventions as Part of Multimodal Treatment Guidelines for the Management of Cervical Radiculopathy: A Delphi Study." Phys Ther.
Triano, J. J., C. M. Rogers, S. Combs, D. Potts and K. Sorrels (2003). "Quantitative feedback versus standard training for cervical and thoracic manipulation." J Manipulative Physiol Ther **26**(3): 131-138.
Tsegay, G. S., G. B. Gebregergs, G. G. Weleslassie and T. T. Hailemariam (2023). "Effectiveness of Thoracic Spine Manipulation on the Management of Neck Pain: A Systematic Review and Meta-Analysis of Randomized Control Trials." J Pain Res **16**: 597-609.
Walker-Bone, K. E., K. T. Palmer, I. Reading and C. Cooper (2003). "Soft-tissue rheumatic disorders of the neck and upper limb: prevalence and risk factors." Semin Arthritis Rheum **33**(3): 185-203.

For peer review only

Yu, H., P. Côté, J. J. Wong, H. M. Shearer, S. Mior, C. Cancelliere, K. Randhawa, A. Ameis, L. J. Carroll, M. Nordin, S. Varatharajan, D. Sutton, D. Southerst, C. Jacobs, M. Stupar, A. Taylor-Vaisey, D. P. Gross, R. J. Brison, M. Paulden, C. Ammendolia, J. D. Cassidy, S. Marshall, R. N. Bohay, J. Stapleton and M. Lacerte (2021). "Noninvasive management of soft tissue disorders of the shoulder: A clinical practice guideline from the Ontario Protocol for Traffic Injury Management (OPTIMa) collaboration." Eur J Pain **25**(8): 1644-1667.
Zunke, P., A. Auffarth, W. Hitzl and M. Moursy (2020). "The effect of manual therapy to the thoracic spine on pain-free grip and sympathetic activity in patients with lateral epicondylalgia humeri. A randomized, sample sized planned, placebo-controlled, patient-blinded monocentric trial." BMC Musculoskelet Disord **21**(1): 186.

For peer review only

PIO: What is the effectiveness of a thoracic high velocity low amplitude thrust (HVLAT) manipulation in patients with musculoskeletal disorders in the upper quadrant, a systematic review.

Walser et al, 2013 retrieved 242 hits and included 13 RCTs in a comparable SR
Ronald F. Walser, Brent B. Meserve & Thomas R. Boucher (2009) The Effectiveness of Thoracic Spine Manipulation for the Management of Musculoskeletal Conditions: A Systematic Review and Meta-Analysis of Randomized Clinical Trials, Journal of Manual & Manipulative Therapy, 17:4, 237-246, DOI: 10.1179/106698109791352085

TOTAL RIS in EndNote: 2075
Double entries: 210
Grey literature / manual searching: 2
Remaining unique manuscripts: 1867
Screening Ti/Ab:
Full Text:
Inclusion for analysis:

OVID search of Embase (146 hits) and Medline (55 hits)

Musculoskeletal adj2 (pain OR disorder* OR disease* OR symptom* OR problem*) OR Neck adj2 (pain OR disorder* OR disease* OR symptom* OR problem*) OR Headache adj2 (disorder* OR tension type OR cervicogenic OR migraine) OR (Cervical radiculopathy) OR (radicular adj2 (syndrome* OR pain)) OR Cervical adj2 (nerve root OR disc OR (disc disease) OR impingement) OR Shoulder adj2 (pain OR disorder* OR disease* OR symptom* OR problem* OR imping*) OR (adhesive capsulitis) OR (frozen shoulder) OR Elbow adj2 (pain OR disorder* OR disease* OR symptom* OR problem* OR tennis) OR epicondylalgia OR epicondylitis OR Temporomandibular adj2 (joint OR pain OR disorder* OR disease* OR symptom* OR problem*) OR Upper extremity adj2 (joint OR pain OR disorder* OR disease* OR symptom* OR problem*) OR Upper quadrant adj2 (joint OR pain OR disorder* OR disease* OR symptom* OR problem*) **n= 71322 / 45490**

AND

Thoracic OR (Thoracic adj2 spine) OR Thora* n= **762664 / 321071**

AND

Spinal manipulation OR Manipulation OR manipulat* OR adjustment OR Chiroprac* OR (high adj2 (velocity OR low OR amplitude OR thrust)) OR treat* OR therapy OR intervention OR SMT OR thrust OR mobilis* OR mobiliz* OR mobilization OR mobilization OR manual therapy OR osteopat* OR physical therapy OR physio* OR rehab* OR management OR HVLA* **n = 17962465 / 13997147**

AND

For peer review only

Pain OR (numerical rating scale) OR NRS OR (numerical pain rating scale) OR NPRS OR Disability OR disable* OR (return to work) OR effectiveness OR effect* OR patient adj2 (reported OR outcome OR measur*) OR (recovery of function) OR activit* adj2 (daily OR living OR life) OR Physical adj2 (examination OR test) OR Range adj2 motion OR Muscle adj2 strength OR Psychosocial* OR (Neck disability index) OR NDI OR (Bournemouth adj2 neck) OR (patient specific functional scale) OR PSFS OR (shoulder pain and disability index) OR SPADI **n = 289510 / 154353**

AND

randomised OR randomized OR random* OR (Clinical trial) OR RCT **n= 3275725 / 1846551**

Web of Science: (1718 hits)

Musculoskeletal NEAR/1 (pain OR disorder* OR disease* OR symptom* OR problem*) OR Neck NEAR/1 (pain OR disorder* OR disease* OR symptom* OR problem*) OR Headache NEAR/1 (disorder* OR “tension type” OR cervicogenic OR migraine) OR (Cervical radiculopathy) OR (radicular NEAR/1 (syndrome* OR pain)) OR Cervical NEAR/1 ((“nerve root” OR disc OR “disc disease”) OR impingement) OR Shoulder NEAR/1 (pain OR disorder* OR disease* OR symptom* OR problem* OR imping*) OR “adhesive capsulitis” OR “frozen shoulder” OR Elbow NEAR/1 (pain OR disorder* OR disease* OR symptom* OR problem* OR tennis) OR epicondylalgia OR epicondylitis OR Temporomandibular NEAR/1 (joint OR pain OR disorder* OR disease* OR symptom* OR problem*) OR Upper extremity NEAR/1 (joint OR pain OR disorder* OR disease* OR symptom* OR problem*) OR Upper quadrant NEAR/2 (joint OR pain OR disorder* OR disease* OR symptom* OR problem*) **n = 124959**

AND

Thoracic OR (Thoracic NEAR/2 spine OR spinal) OR Thoracal **n = 504371**

AND

Spinal manipulation OR Manipulation OR manipulat* OR adjustment OR Chiroprac* OR (high NEAR/1 (velocity OR low OR amplitude OR thrust)) OR treat* OR therapy OR intervention OR smt OR thrust OR mobilis* OR mobiliz* OR mobilization OR mobilization OR manual therapy OR osteopat* OR physical therapy OR physio* OR rehab* OR management OR HVLA* **n = 14369540**

AND

Pain OR measure* OR (numerical rating scale) OR NRS OR (numerical pain rating scale) OR NPRS OR Disability OR disable* OR (return to work) OR patient NEAR/1 (reported OR outcome OR measur*) OR (recovery of function) OR activit* NEAR/1 (daily OR living OR life) OR Physical NEAR/1 (examination OR test) OR Range NEAR/1 motion OR Muscle NEAR/1 strength OR Psychosocial* OR (Neck disability index) OR NDI OR (Bournemouth NEAR/1 neck) OR (patient specific functional scale) OR PSFS OR (shoulder pain and disability index) OR SPADI **n = 22017728**

For peer review only - http://bmjopen.bmj.com/site/about/guidelines.xhtml

AND

randomised OR randomized OR random* OR (Clinical trial) OR RCT **n = 2790253**

SCOPUS [36 hits] also used for CINAHL [17 hits]

Musculoskeletal W/2 pain OR Musculoskeletal W/2 disorder* OR Musculoskeletal W/2 disease* OR Musculoskeletal W/2 symptom* OR Musculoskeletal W/2 problem* OR Neck W/2 pain OR Neck W/2 disorder* OR Neck W/2 disease* OR Neck W/2 symptom* OR Neck W/2 problem* OR Headache W/2 disorder* OR Headache W/2 "tension type" OR Headache W/2 cervicogenic OR Headache W/2 migraine OR "Cervical radiculopathy" OR radicular W/2 syndrome* OR radicular W/2 pain OR radicular W/2 Cervical W/2 "nerve root" OR Cervical W/2 disc OR Cervical W/2 "disc disease" OR Cervical W/2 impingement OR Shoulder W/2 pain OR Shoulder W/2 disorder* OR Shoulder W/2 disease* OR Shoulder W/2 symptom* OR Shoulder W/2 problem* OR Shoulder W/2 imping* OR "adhesive capsulitis" OR "frozen shoulder" OR Elbow W/2 pain OR Elbow W/2 disorder* OR Elbow W/2 disease* OR Elbow W/2 symptom* OR Elbow W/2 problem* OR Elbow W/2 tennis) OR epicondylalgia OR epicondylitis OR Temporomandibular W/2 joint OR Temporomandibular W/2 pain OR Temporomandibular W/2 disorder* OR Temporomandibular W/2 disease* OR Temporomandibular W/2 symptom* OR Temporomandibular W/2 problem* OR "Upper extremity" W/2 joint OR "Upper extremity" pain OR "Upper extremity" disorder* OR "Upper extremity" disease* OR "Upper extremity" symptom* OR "Upper extremity" problem* OR "Upper quadrant" W/2 joint OR "Upper quadrant" W/2 pain OR "Upper quadrant" W/2 disorder* OR "Upper quadrant" W/2 disease* OR "Upper quadrant" W/2 symptom* OR "Upper quadrant" W/2 problem* **n = 27682 in scopus**

AND

Thoracic OR Thoracic W/2 spine OR Thoracic W/2 spinal OR Thora* **n = 330870 in scopus**

AND

"Spinal manipulation" OR Manipulation OR manipulat* OR adjustment OR Chiroprac* OR high W/2 velocity OR high W/2 low OR high W/2 amplitude OR high W/2 thrust OR treat* OR therapy OR intervention OR SMT OR "spinal manipul* therapy" OR thrust OR mobilis* OR mobiliz* OR mobilization OR mobilization OR manual therapy OR osteopat* OR "physical therapy" OR physio* OR rehab* OR management OR HVLA* OR "manipulative technique*" **n = 2512 in scopus**

AND

Pain OR measure* OR "numerical rating scale" OR NRS OR "numerical pain rating scale" OR NPRS OR Disability OR disable* OR "return to work" OR effectiveness OR effect* OR patient W/4 reported OR patient W/4 outcome OR patient W/4 measur* OR "recovery of function" OR activit* W/2 daily OR activit* W/2 living OR activit* W/2 life OR Physical W/2 examination OR Physical W/2 test OR Range W/2 motion OR Muscle W/2 strength OR Psychosocial* OR "neck disability index" OR NDI OR Bournemouth W/2 neck OR Bournemouth W/2 question* OR "patient specific functional scale" OR PSFS OR "shoulder pain and disability index" OR spadi **n = 433039 in scopus**

AND

randomised OR randomized OR random* OR "Clinical trial" OR RCT

For peer review only

Index to Chiropractic Literature [15 hits]

'Thoracic manipulation' AND random*

For peer review only

Reporting checklist for protocol of a systematic review and meta analysis.

Based on the PRISMA-P guidelines.

Instructions to authors

Complete this checklist by entering the page numbers from your manuscript where readers will find each of the items listed below.

Your article may not currently address all the items on the checklist. Please modify your text to include the missing information. If you are certain that an item does not apply, please write "n/a" and provide a short explanation.

Upload your completed checklist as an extra file when you submit to a journal.

In your methods section, say that you used the PRISMA-Preporting guidelines, and cite them as:

Moher D, Shamseer L, Clarke M, Ghersi D, Liberati A, Petticrew M, Shekelle P, Stewart LA. Preferred Reporting Items for Systematic Review and Meta-Analysis Protocols (PRISMA-P) 2015 statement. Syst Rev. 2015;4(1):1.

		Reporting Item	Page Number
**Title**			
Identification	#1a	Identify the report as a protocol of a systematic review	1
Update	#1b	If the protocol is for an update of a previous systematic review, identify as such	n/a

For peer review only

**Registration**			
	#2	If registered, provide the name of the registry (such as PROSPERO) and registration number	1 & 3
**Authors**			
Contact	#3a	Provide name, institutional affiliation, e-mail address of all protocol authors; provide physical mailing address of corresponding author	1
Contribution	#3b	Describe contributions of protocol authors and identify the guarantor of the review	8
**Amendments**			
	#4	If the protocol represents an amendment of a previously completed or published protocol, identify as such and list changes; otherwise, state plan for documenting important protocol amendments	n/a
**Support**			
Sources	#5a	Indicate sources of financial or other support for the review	8
Sponsor	#5b	Provide name for the review funder and / or sponsor	n/a
Role of sponsor or funder	#5c	Describe roles of funder(s), sponsor(s), and / or institution(s), if any, in developing the protocol	n/a
**Introduction**			
Rationale	#6	Describe the rationale for the review in the context of what	2

For peer review only

		is already known	
Objectives	#7	Provide an explicit statement of the question(s) the review will address with reference to participants, interventions, comparators, and outcomes (PICO)	2
**Methods**			
Eligibility criteria	#8	Specify the study characteristics (such as PICO, study design, setting, time frame) and report characteristics (such as years considered, language, publication status) to be used as criteria for eligibility for the review	7
Information sources	#9	Describe all intended information sources (such as electronic databases, contact with study authors, trial registers or other grey literature sources) with planned dates of coverage	7
Search strategy	#10	Present draft of search strategy to be used for at least one electronic database, including planned limits, such that it could be repeated	supplement
Study records - data management	#11a	Describe the mechanism(s) that will be used to manage records and data throughout the review	6
Study records - selection process	#11b	State the process that will be used for selecting studies (such as two independent reviewers) through each phase of the review (that is, screening, eligibility and inclusion in meta-analysis)	5
Study records -	#11c	Describe planned method of extracting data from reports	5

For peer review only

data collection process		(such as piloting forms, done independently, in duplicate), any processes for obtaining and confirming data from investigators	
Data items	#12	List and define all variables for which data will be sought (such as PICO items, funding sources), any pre-planned data assumptions and simplifications	5
Outcomes and prioritization	#13	List and define all outcomes for which data will be sought, including prioritization of main and additional outcomes, with rationale	5
Risk of bias in individual studies	#14	Describe anticipated methods for assessing risk of bias of individual studies, including whether this will be done at the outcome or study level, or both; state how this information will be used in data synthesis	7
Data synthesis	#15a	Describe criteria under which study data will be quantitatively synthesised	5
Data synthesis	#15b	If data are appropriate for quantitative synthesis, describe planned summary measures, methods of handling data and methods of combining data from studies, including any planned exploration of consistency (such as I2, Kendall's τ)	5
Data synthesis	#15c	Describe any proposed additional analyses (such as sensitivity or subgroup analyses, meta-regression)	5
Data synthesis	#15d	If quantitative synthesis is not appropriate, describe the type of summary planned	6

For peer review only

Meta-bias(es)	#16	Specify any planned assessment of meta-bias(es) (such as publication bias across studies, selective reporting within studies)	6
Confidence in cumulative evidence	#17	Describe how the strength of the body of evidence will be assessed (such as GRADE)	6

None The PRISMA-P elaboration and explanation paper is distributed under the terms of the Creative Commons Attribution License CC-BY. This checklist can be completed online using https://www.goodreports.org/, a tool made by the EQUATOR Network in collaboration with Penelope.ai

For peer review only

BMJ Open

Effectiveness of thoracic spine manipulation for upper quadrant musculoskeletal disorders: protocol for a systematic review.

Journal:	*BMJ Open*
Manuscript ID	bmjopen-2023-076143.R1
Article Type:	Protocol
Date Submitted by the Author:	04-Aug-2023
Complete List of Authors:	Thoomes, Erik; University of Birmingham College of Life and Environmental Sciences, School of Sport, Exercise and Rehabilitation Sciences; Fysio-Experts, Research department<br>Tilborghs, Gus; Breederode College, Department of Manual Therapy<br>Heneghan, Nicola; University of Birmingham, School of Sport, Exercise and Rehabilitation Sciences<br>Falla, Deborah; University of Birmingham College of Life and Environmental Sciences, School of Sport, Exercise and Rehabilitation Sciences<br>de Graaf, Marloes; Breederode College, Department of Manual Therapy; Fysio-Experts, Research department
**Primary Subject Heading**:	Rehabilitation medicine
Secondary Subject Heading:	Occupational and environmental medicine
Keywords:	Systematic Review, Spine < ORTHOPAEDIC & TRAUMA SURGERY, Physical Therapy Modalities

SCHOLARONE™
Manuscripts

Effectiveness of thoracic spine manipulation for upper quadrant musculoskeletal disorders: protocol for a systematic review.

ABSTRACT

Introduction

Upper quadrant musculoskeletal disorders (UQMD), comprising of cranial, cervical, shoulder and upper extremity complaints, are among the most frequently reported disorders in clinical practice. Thoracic high velocity low amplitude thrust (Tx-HVLAT) manipulation is a form of conservative management recommended in systematic reviews as an effective treatment option for aspects of UQMD disorders such headache, shoulder pain and lateral elbow pain. However, no recent systematic reviews have assessed the effectiveness across UQMD. Therefore, this systematic review aims to update the current evidence on the effectiveness of Tx-HVLAT for patients with UQMD on 1) patient reported outcomes, 2) performance measures or 3) psychosocial outcomes.

Methods and analysis

Electronic libraries including Cochrane Controlled Trials Register, MEDLINE, EMBASE, CINAHL and Index to Chiropractic Literature will be searched from inception using use MeSH ,Thesaurus and /or free text words. Combinations will be made based on localisation, disorder, intervention and design. Following guidelines as advised by the Cochrane Back Review Group and compliant with PRISMA-P guidelines, published randomised controlled trials will be included

Two review authors will independently assess the risk of bias using the Cochrane Back Review Group's recommended ROB2 tool and will independently extract the data using a standardized data extraction form. Overall quality of the evidence will be evaluated using the GRADE method.

For continuous data, we will calculate standardized mean differences with 95% confidence intervals (SMD; 95%CI). For dichotomous outcomes, Relative Risks (RRs) and 95%CIs will be calculated. Where possible we will present a subgroup analysis by disorder. For pooling a random-effects model will be used.

Ethics and dissemination

The study findings will be submitted to a relevant peer-reviewed journal for dissemination and then presented at relevant conferences. Ethics approval was not required for this systematic review.

Registration details

This systematic review was registered in the PROSPERO database (CRD42023429996).

Strengths and limitations of this study

- This study follows Cochrane Handbook for Systematic Review of Interventions guidelines.
- Risk of bias (ROB) will be assessed using the Cochrane Back Review Group's recommended ROB2 tool.
- This study adheres to PRISMA-P guidelines.

For peer review only

- Strength of the evidence will be evaluated the by using the GRADEpro system.
- A limitation of this study is that results from relevant studies not published in English or Dutch or German might be missed

KEYWORDS
Thoracic spine; spinal manipulation; high velocity low amplitude; effectiveness; systematic review

For peer review only

INTRODUCTION

Upper quadrant musculoskeletal disorders (UQMD), comprising of cranial, cervical, shoulder and upper extremity complaints, are among the most frequently reported disorders in clinical practice (1, 2). They are the second most common cause of work-related musculoskeletal disorders, with only lower back pain being more common (3).

Most clinical guidelines for cervical and upper limb disorders suggest non-surgical management, as a first treatment option for UQMD (4, 5, 6, 7, 8, 9). Spinal manipulation is one such management approach used in clinical practice. Manipulation is defined as "a passive, high velocity, low amplitude thrust (HVLAT) applied to a joint complex within its anatomical limit with the intent to restore optimal motion, function, and/or to reduce pain" in the International Federation of Orthopaedic Manipulative Physical Therapists' (IFOMPT) "Glossary of Terms" (10). Thoracic high velocity low amplitude thrust (Tx-HVLAT) manipulation is an intervention used by both orthopaedic manipulative physical therapists, as well as chiropractors and osteopaths (11, 12). In addition to the emerging evidence supporting its use for UQMD, the thoracic spine is also the most commonly manipulated spinal region (13, 14). One of the reasons for this has been linked to the theory of regional interdependence with the thoracic spine being viewed as a silent contributor to clinical presentations where a pain source lies elsewhere (15).

Tx-HVLAT is a recommended best practice management option for individuals with neck pain (5, 16, 17, 18, 19, 20, 21). Additionally, recent evidence from systematic reviews supports Tx-HVLAT also as an effective treatment option for other upper quadrant disorders such headache (22), shoulder pain (23, 24) and lateral elbow pain (25). However, no recent systematic reviews have assessed the effectiveness across UQMD.

Therefore, this systematic review aims to synthesise the current evidence on the effectiveness of Tx-HVLAT for patients with UQMD on (1) primary outcomes (e.g. pain, disability and perceived effect) and (2) secondary outcomes (e.g. performance measures, psychosocial outcomes, adverse events).

For peer review only

METHODS AND ANALYSIS

Protocol and Registration

This systematic review was registered in the PROSPERO database (CRD42023429996) and written in compliance with PRISMA-2020 guidelines (26).

Selection Criteria

Inclusion criteria

Published randomised clinical trials (RCTs) for which full texts are available in English, Dutch, or German will be included.

Exclusion criteria

Abstracts for which full reports are not available or studies without the outcome of interest will be excluded.

Participants

Adult patients (age ≥ 18 years), with either short-term (less than 3 months), intermediate (3 months to 1 year) or long-term (more than 1 year) UQMD treated in primary care, hospitals educational or occupational settings will be included. For the purpose of this review, UQMD comprises of complaints of pain and/or disability in the head, neck, upper thoracic spine or upper extremity or complaints having been classified or labelled as UQMD .

Interventions

Studies using Tx-HVLAT (with or without cavitation) as an intervention for UQMD will be included. The technique could be provided once or multiple times to a single spinal region or various spinal regions during a single session or over multiple sessions. Co-interventions can also be included within the treatment session if these were also included in the comparison group. This allows for differences in treatment effect to be attributed to the addition of Tx-HVLAT in the experimental group.

Comparison

Comparisons which will be evaluated shall consist of: (a) placebo, waiting list control or no treatment or (b) other type(s) of conservative (i.e. non-surgical) treatment.

Outcome measures

Following guidelines as advised by the Cochrane Back Review Group in establishing our primary outcomes, studies will be included that used at least one of the outcome measures that are considered to be the most important, namely: pain intensity, global perceived effect (e.g. proportion of patients recovered, subjective improvement of symptoms), disability (e.g. Neck Disability Index (NDI), Bournemouth Neck Questionnaire (NBQ), Shoulder Pain and Disability Index (SPADI)), return to work (e.g. days off work) or quality of life (e.g. EQ-5D).

Outcomes of physical examinations (e.g. range of motion, spinal flexibility, muscle strength, upper limb nerve tension testing), and psycho-social outcomes (e.g. anxiety, depression, pain behaviour) will be considered as secondary outcomes. Other outcomes such as drug consumption or adverse side effects will also be considered as secondary outcomes.

Search strategy

The search strategy will follow the recommendation by the Cochrane Handbook for Systematic Review of Interventions (27). Electronic database will be searched from inception and will include the Cochrane Controlled Trials Register, MEDLINE, EMBASE, CINAHL, Index to Chiropractic Literature and the PEDro database. We will use MeSH (Medline), Thesaurus (EMBASE, CINAHL) and free text

For peer review only

words. Combinations will be made based on a) localisation (e.g. head, neck, thoracic spine or upper extremity); b) disorder (e.g. UQMD, headache, neck pain, thoracic spinal pain, shoulder pain, elbow pain or upper extremity disorders); c) intervention (e.g. HVLAT, manipulation, manual therapy, physiotherapy, physical therapy, chiropractic) and d) design: (randomised clinical trial or randomised controlled trial). Manual searches of published review bibliographies and reference lists of primary studies will be undertaken to search for possible studies not captured by the electronic searches (**see appendix 1**).

A research librarian together with a review author (ET) will perform the electronic searches. The search results will be uploaded and managed using EndNote v20 software (Clarivate Analytics, London UK). Two review authors (ET,GT) will independently screen and select potentially eligible studies. Firstly, the title and abstract will be screened for eligibility. Secondly, the full text papers will be assessed to ascertain whether the study meets the inclusion criteria regarding design, participants, and interventions. Disagreements on inclusion will be resolved by discussion or through arbitration by a third review author (MdG).

Risk of bias assessment

Two review authors (ET,GT) will independently assess the risk of bias (ROB) using the Cochrane Back Review Group's recommended ROB2 tool (28). This tool is structured into 5 different domains of bias (arising from the randomisation process, due to deviations from intended interventions, due to missing outcome data, in measurement of the outcome and in selection of the reported result). Within each domain, the assessment comprises: a series of signalling questions, a judgment about risk of bias for the domain, free text boxes to justify responses to the signalling questions and risk-of-bias judgments and optional free text boxes to predict (and explain) the likely direction of bias. When disagreement persists, a third review author (MdG) will be consulted. A low risk of bias is defined as being judged to be at low risk of bias for all domains for the result (29).

Data extraction

Using a standardized data extraction form, two review authors (ET,GT) will independently extract the data (including sample size, participant characteristics, in- & exclusion criteria, type of UQMD, types of interventions and comparators, outcome measures, follow up times and results) of the included RCTs. They will compare their forms and for consistency, precision and accuracy (during a face-to-face meeting) and discuss any discrepancies. In cases of uncertainly about the data extracted, a third review author (MdG) will be consulted.

Data analysis

For continuous data, we will calculate standardized mean differences with 95% confidence intervals (SMD; 95%CI). SMD will be used because different measures are frequently used to address the same clinical outcome. Where applicable, the weighted mean difference (WMD) will be calculated. All data from

For peer review only

Visual Analogue Scales (VAS) or Numerical Rating Scales (NRS) will be converted to scales ranging from 0-100, where necessary. For dichotomous outcomes, Relative Risks (RRs) and 95% Confidence Interval (CI) will be calculated. If the published article does not provide enough data, we will contact the original authors in an effort to retrieve additional necessary data, with a reminder being sent after two weeks.

Where possible we will present a subgroup analysis by disorder (e.g. neck pain, shoulder pain, headache). Prior to pooling, clinical heterogeneity sources such as differences in participant characteristics (e.g., age, baseline disease severity, ethnicity, comorbidities), types or timing of outcome measurements and intervention characteristics (e.g., dose, frequency of dose, training of interventionists) will be assessed through discussion with the research team (30). If the research team decides pooling is appropriate, sub-grouping and meta-analysis will be considered. For pooling a random-effects model will be used (31). If multiple time points of outcome are reported, we will report (i) immediate, (ii) closest to 6 weeks, and (iii) closets to 3 months).

RevMan Analyses (RevMan 5.3) will be used to analyse the data. The inter-observer reliability of the risk of bias assessments will be calculated using Kappa and categorised agreement as poor (0.0), slight (0.0-0.2), fair (0.21-0.4), moderate (0.41-0.6), substantial (0.61-0.8), or almost perfect (0.81-1.0) (32).

Strength of the evidence

The overall quality of the evidence will be evaluated using the GRADE method (33) . The quality of the evidence will be based upon five principal factors: (1) limitations in study design (downgraded when >25% of the participants are from studies with a high risk of bias), (2) inconsistency of results [downgraded when there is statistical heterogeneity (I^2 >40%) or inconsistent findings (defined as ≤75% of the participants reporting findings in the same direction)], (3) indirectness (e.g. generalizability of the findings), (4) imprecision (downgraded when the total number of participants across studies is <300 for each outcome) and (5) other considerations, such as reporting bias. The quality of the evidence will be downgraded by one level when one of the factors described above is met (29, 34).

Two independent reviewers (ET,GT) will grade the quality of evidence. Single studies will be considered inconsistent and imprecise (i.e. sparse data) and providing ‘‘low quality evidence’’, which can be further downgraded to ‘‘very low quality evidence’’ if there are also limitations in design or indirectness. The following grading of quality of the evidence will be applied (33):

- High quality evidence: further research is very unlikely to change confidence in estimate of effect.
- Moderate quality evidence: further research is likely to have an important impact on confidence in estimate of effect and may change the estimate.
- Low quality evidence: further research is very likely to have an important impact on confidence in estimate of effect and is likely to change the estimate.
- Very low quality evidence: very little confidence in the effect estimate.
- No evidence: no RCTs were identified that addressed this outcome

For peer review only

Patient and public involvement

The research question in this study forms part of a larger discussion within our patient and public involvement meetings as part of an existing programme of a multi-centre research programme that is focused on UQMD. Patients will not be involved in the analysis and data collection of the study.

Ethics and dissemination

Ethics approval was not required for the systematic review. The study findings will be submitted to a relevant peer-reviewed journal for dissemination and then presented at relevant conferences.

Strengths and limitations of this study

- This study follows Cochrane Handbook for Systematic Review of Interventions guidelines.
- Risk of bias (ROB) will be assessed using the Cochrane Back Review Group's recommended ROB2 tool.
- This study adheres to PRISMA-P guidelines.
- Strength of the evidence will be evaluated the by using the GRADEpro system.
- A limitation of this study is that results from relevant studies not published in English or Dutch or German might be missed

DISCUSSION

Thoracic spine manipuation (Tx-HVLAT) is reported to be one of the most often used treatment modality in multi-modal management strategies, not only for thoracic spinal pain but also for many other upper quandrant musculoskeletal disorders (UQMD). Findings from this study may assist clinicians and researchers in formulating an individualised management plan for patients with UQMD. By assessing the effectiveness of Tx-HVLAT for different UQMD, clinicians will be better able to choose when to incorporate Tx-HVLAT as an effective treatment modality in evidence based multi-modal management strategies, instead of using a standardised 'one size fits all' approach. Findings from this study will also serve a need both clinically and within the contemporary literature to inform further research (e.g. on efficacy, cost effectiveness, etc.). We also aim to compare and contrast this study's findings with previously published systematic reviews (18, 19, 20, 21, 22, 24). While results from relevant studies not published in English or Dutch or German might not be analyzed, we feel this will not impact the final outcome as it has been reported that exclusion of trials reported in a language other than English does not significantly affect the results of meta-analyses (35).

Twitter

Erik Thoomes @Fysio_Experts

For peer review only

Deborah Falla @Deb_Falla
Nicola Heneghan @HeneghanNicola

Author contributions

MdG devised the initial focus of this systematic review with the help of all authors. ET and GT are PGR students, MdG is the lead supervisor, NH and DF are co-researchers. ET drafted the initial protocol manuscript with lead and co-supervisors providing guidance on methodological decisions and proposed analyses. All authors have contributed subject-specific expertise. All authors will contribute to data interpretation, conclusions and dissemination. All authors have read, contributed to and agreed to the final manuscript. DF and MdG are both guarantors of the study.

Funding

This study will be conducted as part of a Post Graduate Research project though the University of Birmingham, UK and as such received no specific grant from any funding agency in the public, commercial or not-for-profit sectors.

Competing interests

None declared.

Patient consent for publication

Not required.

Provenance and peer review

Not commissioned; externally peer reviewed.

Open access

This is an open access article distributed in accordance with the Creative Commons Attribution Non Commercial (CC BY-NC 4.0) license, which permits others to distribute, remix, adapt, build upon this work non-commercially, and license their derivative works on different terms, provided the original work is properly cited, appropriate credit is given, any changes made indicated, and the use is non-commercial.

Data statement section

Technical appendix, statistical code, and dataset available from the University of Birmingham eData Repository available at: https://edata.bham.ac.uk/

For peer review only

REFERENCES

1. Huisstede BM, Bierma-Zeinstra SM, Koes BW, Verhaar JA. Incidence and prevalence of upper-extremity musculoskeletal disorders. A systematic appraisal of the literature. BMC musculoskeletal disorders. 2006;7:7.
2. Walker-Bone KE, Palmer KT, Reading I, Cooper C. Soft-tissue rheumatic disorders of the neck and upper limb: prevalence and risk factors. Semin Arthritis Rheum. 2003;33(3):185-203.
3. Executive H. Work-related Musculoskeletal Disorders (WRMSDs) Statistics in Great Britain. 2017. 2016.
4. Bier JD, Scholten-Peeters WGM, Staal JB, Pool J, van Tulder MW, Beekman E, et al. Clinical Practice Guideline for Physical Therapy Assessment and Treatment in Patients With Nonspecific Neck Pain. Phys Ther. 2018;98(3):162-71.
5. Blanpied PR, Gross AR, Elliott JM, Devaney LL, Clewley D, Walton DM, et al. Neck Pain: Revision 2017. The Journal of orthopaedic and sports physical therapy. 2017;47(7):A1-a83.
6. Lafrance S, Charron M, Roy JS, Dyer JO, Frémont P, Dionne CE, et al. Diagnosing, Managing, and Supporting Return to Work of Adults With Rotator Cuff Disorders: A Clinical Practice Guideline. The Journal of orthopaedic and sports physical therapy. 2022;52(10):647-64.
7. Lapner P, Alfonso A, Herbert-Davies J, Pollock JW, Marsh J, King G. Position statement: nonoperative management of lateral epicondylitis in adults. Can J Surg. 2022;65(5):E625-e9.
8. Yu H, Côté P, Wong JJ, Shearer HM, Mior S, Cancelliere C, et al. Noninvasive management of soft tissue disorders of the shoulder: A clinical practice guideline from the Ontario Protocol for Traffic Injury Management (OPTIMa) collaboration. European journal of pain (London, England). 2021;25(8):1644-67.
9. Hegmann KT, Hoffman HE, Belcourt RM, Byrne K, Glass L, Melhorn JM, et al. ACOEM practice guidelines: elbow disorders. J Occup Environ Med. 2013;55(11):1365-74.
10. Rusthon A ea. The Standards Document, Glossary of Terms: IFOMPT; 2012 [Available from: http://www.ifompt.com/Standards/SC+Glossary.html.
11. Heneghan NR, Puentedura EJ, Arranz I, Rushton A. Thoracic thrust joint manipulation: An international survey of current practice and knowledge in IFOMPT member countries. Musculoskeletal science & practice. 2020;50:102251.
12. Triano JJ, Rogers CM, Combs S, Potts D, Sorrels K. Quantitative feedback versus standard training for cervical and thoracic manipulation. Journal of manipulative and physiological therapeutics. 2003;26(3):131-8.
13. Adams G, Sim J. A survey of UK manual therapists' practice of and attitudes towards manipulation and its complications. Physiotherapy research international : the journal for researchers and clinicians in physical therapy. 1998;3(3):206-27.
14. Puentedura EJ, Slaughter R, Reilly S, Ventura E, Young D. Thrust joint manipulation utilization by U.S. physical therapists. The Journal of manual & manipulative therapy. 2017;25(2):74-82.
15. Heneghan NR, Rushton A. Understanding why the thoracic region is the 'Cinderella' region of the spine. Man Ther. 2016;21:274-6.
16. Côté P, Wong JJ, Sutton D, Shearer HM, Mior S, Randhawa K, et al. Management of neck pain and associated disorders: A clinical practice guideline from the Ontario Protocol for Traffic Injury Management (OPTIMa) Collaboration. European Spine Journal. 2016;25(7):2000-22.
17. Thoomes E, Graaf MT, Cleland JA, Gallina A, Falla D. Timing of Evidence-Based Nonsurgical Interventions as Part of Multimodal Treatment Guidelines for the Management of Cervical Radiculopathy: A Delphi Study. Phys Ther. 2022.
18. Cross KM, Kuenze C, Grindstaff TL, Hertel J. Thoracic spine thrust manipulation improves pain, range of motion, and self-reported function in patients with mechanical neck

For peer review only

pain: a systematic review. The Journal of orthopaedic and sports physical therapy. 2011;41(9):633-42.
19. Huisman PA, Speksnijder CM, de Wijer A. The effect of thoracic spine manipulation on pain and disability in patients with non-specific neck pain: a systematic review. Disabil Rehabil. 2013;35(20):1677-85.
20. Masaracchio M, Kirker K, States R, Hanney WJ, Liu X, Kolber M. Thoracic spine manipulation for the management of mechanical neck pain: A systematic review and meta-analysis. PloS one. 2019;14(2):e0211877.
21. Tsegay GS, Gebregergs GB, Weleslassie GG, Hailemariam TT. Effectiveness of Thoracic Spine Manipulation on the Management of Neck Pain: A Systematic Review and Meta-Analysis of Randomized Control Trials. Journal of pain research. 2023;16:597-609.
22. Bini P, Hohenschurz-Schmidt D, Masullo V, Pitt D, Draper-Rodi J. The effectiveness of manual and exercise therapy on headache intensity and frequency among patients with cervicogenic headache: a systematic review and meta-analysis. Chiropractic & manual therapies. 2022;30(1):49.
23. Strunce JB, Walker MJ, Boyles RE, Young BA. The immediate effects of thoracic spine and rib manipulation on subjects with primary complaints of shoulder pain. The Journal of manual & manipulative therapy. 2009;17(4):230-6.
24. Sanzo P, Yeung E, Levesque L, Maheu E, Woodard T, Michels S, et al. Is thoracic manipulation effective in managing shoulder dysfunction–A systematic review. Man Ther. 2016;100(25):e154.
25. Zunke P, Auffarth A, Hitzl W, Moursy M. The effect of manual therapy to the thoracic spine on pain-free grip and sympathetic activity in patients with lateral epicondylalgia humeri. A randomized, sample sized planned, placebo-controlled, patient-blinded monocentric trial. BMC musculoskeletal disorders. 2020;21(1):186.
26. Page MJ, McKenzie JE, Bossuyt PM, Boutron I, Hoffmann TC, Mulrow CD, et al. The PRISMA 2020 statement: an updated guideline for reporting systematic reviews. BMJ (Clinical research ed). 2021;372:n71.
27. Higgins JP, Thomas J, Chandler J, Cumpston M, Li T, Page MJ, et al. Cochrane handbook for systematic reviews of interventions: John Wiley & Sons; 2019.
28. Sterne JAC, Savović J, Page MJ, Elbers RG, Blencowe NS, Boutron I, et al. RoB 2: a revised tool for assessing risk of bias in randomised trials. BMJ (Clinical research ed). 2019;366:l4898.
29. Atkins D, Best D, Briss PA, Eccles M, Falck-Ytter Y, Flottorp S, et al. Grading quality of evidence and strength of recommendations. BMJ (Clinical research ed). 2004;328(7454):1490.
30. Higgins JP, Thomas J, Chandler J, Cumpston M, Li T, Page MJ, et al. Cochrane handbook for systematic reviews of interventions; v 6.3: John Wiley & Sons; 2022.
31. Dettori JR, Norvell DC, Chapman JR. Fixed-Effect vs Random-Effects Models for Meta-Analysis: 3 Points to Consider. Global spine journal. 2022;12(7):1624-6.
32. Landis JR, Koch GG. The measurement of observer agreement for categorical data. Biometrics. 1977;33(1):159-74.
33. GRADE-Pro. GRADE handbook for grading quality of evidence and strength of recommendations. Updated. Schünemann H BJ, Guyatt G, Oxman A, editors, editor: The GRADE Working Group; 2013.
34. Brozek JL, Akl EA, Alonso-Coello P, Lang D, Jaeschke R, Williams JW, et al. Grading quality of evidence and strength of recommendations in clinical practice guidelines. Part 1 of 3. An overview of the GRADE approach and grading quality of evidence about interventions. Allergy. 2009;64(5):669-77.
35. Moher D, Pham B, Lawson ML, Klassen TP. The inclusion of reports of randomised trials published in languages other than English in systematic reviews. Health technology assessment (Winchester, England). 2003;7(41):1-90.

For peer review only

PIO: What is the effectiveness of a thoracic high velocity low amplitude thrust (HVLAT) manipulation in patients with musculoskeletal disorders in the upper quadrant, a systematic review.

OVID search of Embase and Medline

Musculoskeletal adj2 (pain OR disorder* OR disease* OR symptom* OR problem*) OR Neck adj2 (pain OR disorder* OR disease* OR symptom* OR problem*) OR Headache adj2 (disorder* OR tension type OR cervicogenic OR migraine) OR (Cervical radiculopathy) OR (radicular adj2 (syndrome* OR pain)) OR Cervical adj2 (nerve root OR disc OR (disc disease) OR impingement) OR Shoulder adj2 (pain OR disorder* OR disease* OR symptom* OR problem* OR imping*) OR (adhesive capsulitis) OR (frozen shoulder) OR Elbow adj2 (pain OR disorder* OR disease* OR symptom* OR problem* OR tennis) OR epicondylalgia OR epicondylitis OR Temporomandibular adj2 (joint OR pain OR disorder* OR disease* OR symptom* OR problem*) OR Upper extremity adj2 (joint OR pain OR disorder* OR disease* OR symptom* OR problem*) OR Upper quadrant adj2 (joint OR pain OR disorder* OR disease* OR symptom* OR problem*)

AND

Thoracic OR (Thoracic adj2 spine) OR Thora*

AND

Spinal manipulation OR Manipulation OR manipulat* OR adjustment OR Chiroprac* OR (high adj2 (velocity OR low OR amplitude OR thrust)) OR treat* OR therapy OR intervention OR SMT OR thrust OR mobilis* OR mobiliz* OR mobilization OR mobilization OR manual therapy OR osteopat* OR physical therapy OR physio* OR rehab* OR management OR HVLA*

AND

Pain OR (numerical rating scale) OR NRS OR (numerical pain rating scale) OR NPRS OR Disability OR disable* OR (return to work) OR effectiveness OR effect* OR patient adj2 (reported OR outcome OR measur*) OR (recovery of function) OR activit* adj2 (daily OR living OR life) OR Physical adj2 (examination OR test) OR Range adj2 motion OR Muscle adj2 strength OR Psychosocial* OR (Neck disability index) OR NDI OR (Bournemouth adj2 neck) OR (patient specific functional scale) OR PSFS OR (shoulder pain and disability index) OR SPADI
AND

For peer review only

Web of Science:

Musculoskeletal NEAR/1 (pain OR disorder* OR disease* OR symptom* OR problem*) OR Neck NEAR/1 (pain OR disorder* OR disease* OR symptom* OR problem*) OR Headache NEAR/1 (disorder* OR “tension type” OR cervicogenic OR migraine) OR (Cervical radiculopathy) OR (radicular NEAR/1 (syndrome* OR pain)) OR Cervical NEAR/1 ((“nerve root” OR disc OR “disc disease”) OR impingement) OR Shoulder NEAR/1 (pain OR disorder* OR disease* OR symptom* OR problem* OR imping*) OR “adhesive capsulitis” OR “frozen shoulder” OR Elbow NEAR/1 (pain OR disorder* OR disease* OR symptom* OR problem* OR tennis) OR epicondylalgia OR epicondylitis OR Temporomandibular NEAR/1 (joint OR pain OR disorder* OR disease* OR symptom* OR problem*) OR Upper extremity NEAR/1 (joint OR pain OR disorder* OR disease* OR symptom* OR problem*) OR Upper quadrant NEAR/2 (joint OR pain OR disorder* OR disease* OR symptom* OR problem*)

AND

Thoracic OR (Thoracic NEAR/2 spine OR spinal) OR Thoracal

AND

Spinal manipulation OR Manipulation OR manipulat* OR adjustment OR Chiroprac* OR (high NEAR/1 (velocity OR low OR amplitude OR thrust)) OR treat* OR therapy OR intervention OR smt OR thrust OR mobilis* OR mobiliz* OR mobilization OR mobilization OR manual therapy OR osteopat* OR physical therapy OR physio* OR rehab* OR management OR HVLA*

AND

Pain OR measure* OR (numerical rating scale) OR NRS OR (numerical pain rating scale) OR NPRS OR Disability OR disable* OR (return to work) OR patient NEAR/1 (reported OR outcome OR measur*) OR (recovery of function) OR activit* NEAR/1 (daily OR living OR life) OR Physical NEAR/1 (examination OR test) OR Range NEAR/1 motion OR Muscle NEAR/1 strength OR Psychosocial* OR (Neck disability index) OR NDI OR (Bournemouth NEAR/1 neck) OR (patient specific functional scale) OR PSFS OR (shoulder pain and disability index) OR SPADI

AND

randomised OR randomized OR random* OR (Clinical trial) OR RCT

SCOPUS; also used for CINAHL

Musculoskeletal W/2 pain OR Musculoskeletal W/2 disorder* OR Musculoskeletal W/2 disease* OR Musculoskeletal W/2 symptom* OR Musculoskeletal W/2 problem* OR Neck W/2 pain OR Neck W/2 disorder* OR Neck W/2 disease* OR Neck W/2 symptom* OR Neck W/2 problem* OR Headache W/2 disorder* OR Headache W/2 “tension type” OR Headache W/2 cervicogenic OR Headache W/2 migraine OR “Cervical radiculopathy” OR radicular W/2 syndrome* OR radicular W/2 pain OR radicular W/2 Cervical W/2 “nerve root” OR Cervical W/2 disc OR Cervical W/2 “disc disease” OR Cervical W/2 impingement OR Shoulder W/2 pain OR Shoulder W/2 disorder* OR Shoulder W/2 disease* OR Shoulder W/2 symptom* OR Shoulder W/2 problem* OR Shoulder W/2 imping* OR “adhesive capsulitis” OR “frozen shoulder” OR Elbow W/2

For peer review only

pain OR Elbow W/2 disorder* OR Elbow W/2 disease* OR Elbow W/2 symptom* OR Elbow W/2 problem* OR Elbow W/2 tennis) OR epicondylalgia OR epicondylitis OR Temporomandibular W/2 joint OR Temporomandibular W/2 pain OR Temporomandibular W/2 disorder* OR Temporomandibular W/2 disease* OR Temporomandibular W/2 symptom* OR Temporomandibular W/2 problem* OR "Upper extremity" W/2 joint OR "Upper extremity" pain OR "Upper extremity" disorder* OR "Upper extremity" disease* OR "Upper extremity" symptom* OR "Upper extremity" problem* OR "Upper quadrant" W/2 joint OR "Upper quadrant" W/2 pain OR "Upper quadrant" W/2 disorder* OR "Upper quadrant" W/2 disease* OR "Upper quadrant" W/2 symptom* OR "Upper quadrant" W/2 problem*

AND

Thoracic OR Thoracic W/2 spine OR Thoracic W/2 spinal OR Thora*

AND

"Spinal manipulation" OR Manipulation OR manipulat* OR adjustment OR Chiroprac* OR high W/2 velocity OR high W/2 low OR high W/2 amplitude OR high W/2 thrust OR treat* OR therapy OR intervention OR SMT OR "spinal manipul* therapy" OR thrust OR mobilis* OR mobiliz* OR mobilization OR mobilization OR manual therapy OR osteopat* OR "physical therapy" OR physio* OR rehab* OR management OR HVLA* OR "manipulative technique*"

AND

Pain OR measure* OR "numerical rating scale" OR NRS OR "numerical pain rating scale" OR NPRS OR Disability OR disable* OR "return to work" OR effectiveness OR effect* OR patient W/4 reported OR patient W/4 outcome OR patient W/4 measur* OR "recovery of function" OR activit* W/2 daily OR activit* W/2 living OR activit* W/2 life OR Physical W/2 examination OR Physical W/2 test OR Range W/2 motion OR Muscle W/2 strength OR Psychosocial* OR "neck disability index" OR NDI OR Bournemouth W/2 neck OR Bournemouth W/2 question* OR "patient specific functional scale" OR PSFS OR "shoulder pain and disability index" OR spadi

AND

randomised OR randomized OR random* OR "Clinical trial" OR RCT

Index to Chiropractic Literature

'Thoracic manipulation' AND random*

PEDro database

Thorac* AND manip*

For peer review only

BMJ Open

Effectiveness of thoracic spine manipulation for upper quadrant musculoskeletal disorders: protocol for a systematic review

Journal:	*BMJ Open*
Manuscript ID	bmjopen-2023-076143.R2
Article Type:	Protocol
Date Submitted by the Author:	31-Aug-2023

Complete List of Authors:	Thoomes, Erik; University of Birmingham College of Life and Environmental Sciences, School of Sport, Exercise and Rehabilitation Sciences; Fysio-Experts, Research department<br>Tilborghs, Gus; Breederode College, Department of Manual Therapy<br>Heneghan, Nicola; University of Birmingham, School of Sport, Exercise and Rehabilitation Sciences<br>Falla, Deborah; University of Birmingham College of Life and Environmental Sciences, School of Sport, Exercise and Rehabilitation Sciences<br>de Graaf, Marloes; Breederode College, Department of Manual Therapy; Fysio-Experts, Research department

**Primary Subject Heading**:	Rehabilitation medicine
Secondary Subject Heading:	Occupational and environmental medicine
Keywords:	Systematic Review, Spine < ORTHOPAEDIC & TRAUMA SURGERY, Physical Therapy Modalities

SCHOLARONE™
Manuscripts

Effectiveness of thoracic spine manipulation for upper quadrant musculoskeletal disorders: protocol for a systematic review

Erik Thoomes [1, 2], Gus Tilborghs [3], Nicola R Heneghan [1], Deborah Falla [1], Marloes de Graaf [2,3]

[1]Centre of Precision Rehabilitation for Spinal Pain (CPR Spine), School of Sport, Exercise and Rehabilitation Sciences, College of Life and Environmental Sciences, University of Birmingham, Birmingham. United Kingdom
[2] Fysio-Experts, Research department, Hazerswoude, The Netherlands
[3] Department of Manual Therapy, Breederode University of Applied Science, Rotterdam, the Netherlands

Word count: 2034

Correspondence to:
Erik Thoomes
erikthoomes@gmail.com

Email addresses and ORCID iDs
Erik Thoomes: erikthoomes@gmail.com 0000-0001-6375-2267
Marloes de Graaf: marloesdegraaf@icloud.com 0000-0002-2865-5389
Nicola Heneghan: n.heneghan@bham.ac.uk 0000-0001-7599-3674
Deborah Falla: d.falla@bham.ac.uk 0000-0003-1689-6190
Gus Tilborghs: gusdevlieger@msn.com NA

ABSTRACT

Introduction

Upper quadrant musculoskeletal disorders (UQMD), comprising of cranial, cervical, shoulder and upper extremity complaints, are among the most frequently reported disorders in clinical practice. Thoracic high velocity low amplitude thrust (Tx-HVLAT) manipulation is a form of conservative management recommended in systematic reviews as an effective treatment option for aspects of UQMD disorders such headache, shoulder pain and lateral elbow pain. However, no recent systematic reviews have assessed the effectiveness across UQMD. Therefore, this systematic review aims to update the current evidence on the effectiveness of Tx-HVLAT for patients with UQMD on 1) patient reported outcomes, 2) performance measures or 3) psychosocial outcomes.

Methods and analysis

The Cochrane Controlled Trials Register, MEDLINE, EMBASE, CINAHL, PEDro and Index to Chiropractic Literature will be searched from inception using use MeSH, Thesaurus and/or free text words. Combinations will be made based on localisation, disorder, intervention and design. Following guidelines as advised by the Cochrane Back Review Group, published randomised controlled trials will be included. Two

For peer review only

review authors will independently assess the risk of bias using the Cochrane Back Review Group's recommended ROB2 tool and will independently extract the data using a standardized data extraction form. Overall quality of the evidence will be evaluated using the GRADE method. For continuous data, we will calculate standardized mean differences with 95% confidence intervals (SMD; 95%CI). For dichotomous outcomes, Relative Risks (RRs) and 95%CIs will be calculated. Where possible we will present a subgroup analysis by disorder. For pooling, a random-effects model will be used.

Ethics and dissemination

Ethics approval is not required for this systematic review. The study findings will be submitted to a relevant peer-reviewed journal for dissemination and presented at relevant conferences.

Study registration

PROSPERO, CRD42023429996.

Strengths and limitations of this study

- This study follows Cochrane Handbook for Systematic Review of Interventions guidelines.
- Risk of bias (ROB) will be assessed using the Cochrane Back Review Group's recommended ROB2 tool.
- Strength of the evidence will be evaluated the by using the GRADEpro system.
- A limitation of the study is that results from relevant studies not published in English, Dutch or German could be missed.

KEYWORDS

Thoracic spine; spinal manipulation; high velocity low amplitude; effectiveness; systematic review

For peer review only

INTRODUCTION

Upper quadrant musculoskeletal disorders (UQMD), comprising of cranial, cervical, shoulder and upper extremity complaints, are among the most frequently reported disorders in clinical practice (1, 2). They are the second most common cause of work-related musculoskeletal disorders, with only lower back pain being more common (3).

Most clinical guidelines for cervical and upper limb disorders suggest non-surgical management, as a first treatment option for UQMD (4, 5, 6, 7, 8, 9). Spinal manipulation is one such management approach used in clinical practice. Manipulation is defined as "a passive, high velocity, low amplitude thrust (HVLAT) applied to a joint complex within its anatomical limit with the intent to restore optimal motion, function, and/or to reduce pain" in the International Federation of Orthopaedic Manipulative Physical Therapists' (IFOMPT) "Glossary of Terms" (10). Thoracic high velocity low amplitude thrust (Tx-HVLAT) manipulation is an intervention used by both orthopaedic manipulative physical therapists, as well as chiropractors and osteopaths (11, 12). In addition to the emerging evidence supporting its use for UQMD, the thoracic spine is also the most commonly manipulated spinal region (13, 14). One of the reasons for this has been linked to the theory of regional interdependence with the thoracic spine being viewed as a silent contributor to clinical presentations where a pain source lies elsewhere (15).

Tx-HVLAT is a recommended best practice management option for individuals with neck pain (5, 16, 17, 18, 19, 20, 21). Additionally, recent evidence from systematic reviews supports Tx-HVLAT also as an effective treatment option for other upper quadrant disorders such headache (22), shoulder pain (23, 24) and lateral elbow pain (25). However, no recent systematic reviews have assessed the effectiveness across UQMD.

Therefore, this systematic review aims to synthesise the current evidence on the effectiveness of Tx-HVLAT for patients with UQMD on (1) primary outcomes (e.g. pain, disability and perceived effect) and (2) secondary outcomes (e.g. performance measures, psychosocial outcomes, adverse events).

For peer review only

METHODS AND ANALYSIS

Protocol and registration

This systematic review was registered in the PROSPERO database (CRD42023429996) and this protocol was prepared following PRISMA-P guidance. The results will be reported in compliance with PRISMA-2020 guidelines (26).

Selection criteria

Inclusion criteria

Published randomised clinical trials (RCTs) for which full texts are available in English, Dutch, or German will be included.

Exclusion criteria

Abstracts for which full reports are not available or studies without the outcome of interest will be excluded.

Participants

Adult patients (age ≥ 18 years), with either short-term (less than 3 months), intermediate (3 months to 1 year) or long-term (more than 1 year) UQMD treated in primary care, hospitals educational or occupational settings will be included. For the purpose of this review, UQMD comprises of complaints of pain and/or disability in the head, neck, upper thoracic spine or upper extremity or complaints having been classified or labelled as UQMD.

Interventions

Studies using Tx-HVLAT (with or without cavitation) as an intervention for UQMD will be included. The technique could be provided once or multiple times to a single spinal region or various spinal regions during a single session or over multiple sessions. Co-interventions can also be included within the treatment session if these were also included in the comparison group. This allows for differences in treatment effect to be attributed to the addition of Tx-HVLAT in the experimental group.

Comparison

Comparisons which will be evaluated shall consist of: (a) placebo, sham manipulation, waiting list control or no treatment or (b) other type(s) of conservative (i.e. non-surgical) treatment.

Outcome measures

Following guidelines as advised by the Cochrane Back Review Group in establishing our primary outcomes, studies will be included that used at least one of the outcome measures that are considered to be the most important, namely: pain intensity, global perceived effect (e.g. proportion of patients recovered, subjective improvement of symptoms), disability (e.g. Neck Disability Index (NDI), Bournemouth Neck Questionnaire (NBQ), Shoulder Pain and Disability Index (SPADI)), return to work (e.g. days off work) or quality of life (e.g. EQ-5D).

Outcomes of physical examinations (e.g. range of motion, spinal flexibility, muscle strength, upper limb nerve tension testing), and psycho-social outcomes (e.g. anxiety, depression, pain behaviour) will be considered as secondary outcomes. Other outcomes such as drug consumption or adverse side effects will also be considered as secondary outcomes.

Search strategy

The search strategy will follow the recommendation by the Cochrane Handbook for Systematic Review of Interventions (27). The following electronic databases will be searched from inception: the Cochrane Controlled Trials Register, MEDLINE, EMBASE, CINAHL, Index to Chiropractic Literature and the PEDro database. Only studies of which the full text is available in English, German or Dutch language will be

For peer review only

included. We will use MeSH (Medline), Thesaurus (EMBASE, CINAHL) and free text words. Combinations will be made based on a) localisation (e.g. head, neck, thoracic spine or upper extremity); b) disorder (e.g. UQMD, headache, neck pain, thoracic spinal pain, shoulder pain, elbow pain or upper extremity disorders); c) intervention (e.g. HVLAT, manipulation, manual therapy, physiotherapy, physical therapy, chiropractic) and d) design: (randomised clinical trial or randomised controlled trial). Manual searches of published review bibliographies and reference lists of primary studies will be undertaken to search for possible studies not captured by the electronic searches. For the search strategies, please refer to **Appendix 1**.

A research librarian together with a review author (ET) will perform the electronic searches. The search results will be uploaded and managed using EndNote v20 software (Clarivate Analytics, London UK). Two review authors (ET, GT) will independently screen and select potentially eligible studies. Firstly, the title and abstract will be screened for eligibility. Secondly, the full text papers will be assessed to ascertain whether the study meets the inclusion criteria regarding design, participants, and interventions. Disagreements on inclusion will be resolved by discussion or through arbitration by a third review author (MdG).

Risk of bias assessment

Two review authors (ET, GT) will independently assess the risk of bias (ROB) using the Cochrane Back Review Group's recommended ROB2 tool (28). This tool is structured into 5 different domains of bias (arising from the randomisation process, due to deviations from intended interventions, due to missing outcome data, in measurement of the outcome and in selection of the reported result). Within each domain, the assessment comprises: a series of signalling questions, a judgment about risk of bias for the domain, free text boxes to justify responses to the signalling questions and risk-of-bias judgments and optional free text boxes to predict (and explain) the likely direction of bias. In case of a "sham" manipulation being the comparator, in the ROB assessment 2.1 this item will be rated as "perhaps" in cases where the authors do not explicitly show that participants were unaware of their assigned intervention. When disagreement persists, a third review author (MdG) will be consulted. A low risk of bias is defined as being judged to be at low risk of bias for all domains for the result (29).

Data extraction

Using a standardized data extraction form, two review authors (ET, GT) will independently extract the data (including sample size, participant characteristics, in- & exclusion criteria, type of UQMD, types of interventions and comparators, outcome measures, follow up times and results) of the included RCTs. They will compare extractions in a face-to-face meeting. In cases of uncertainly about the data extracted, a third review author (MdG) will be consulted.

Data analysis

For peer review only

For continuous data, we will calculate standardized mean differences with 95% confidence intervals (SMD; 95%CI). SMD will be used because different measures are frequently used to address the same clinical outcome. Where applicable, the weighted mean difference (WMD) will be calculated. All data from Visual Analogue Scales (VAS) or Numerical Rating Scales (NRS) will be converted to scales ranging from 0-100, where necessary. For dichotomous outcomes, Relative Risks (RRs) and 95% Confidence Interval (CI) will be calculated. If the published article does not provide enough data, we will contact the original authors in an effort to retrieve additional necessary data, with a reminder being sent after two weeks.

Where possible we will present a subgroup analysis by disorder (e.g. neck pain, shoulder pain, headache) and by comparator intervention. Prior to pooling, clinical heterogeneity sources such as differences in participant characteristics (e.g., age, baseline disease severity, ethnicity, comorbidities), types or timing of outcome measurements and intervention characteristics (e.g., dose, frequency of dose, training of interventionists) will be assessed through discussion with the research team (30). If the research team decides pooling is appropriate, sub-grouping and meta-analysis will be considered. For pooling a random-effects model will be used (31). If multiple time points of outcome are reported, we will report (i) immediate, (ii) closest to 6 weeks, and (iii) closests to 3 months.

RevMan Analyses (RevMan 5.3) will be used to analyse the data. The inter-observer reliability of the risk of bias assessments will be calculated using Kappa and categorised agreement as poor (0.0), slight (0.0-0.2), fair (0.21-0.4), moderate (0.41-0.6), substantial (0.61-0.8), or almost perfect (0.81-1.0) (32).

Strength of the evidence

The overall quality of the evidence will be evaluated using the GRADE method (33) . The quality of the evidence will be based upon five principal factors: (1) limitations in study design (downgraded when >25% of the participants are from studies with a high risk of bias), (2) inconsistency of results [downgraded when there is statistical heterogeneity (I^2 >40%) or inconsistent findings (defined as ≤75% of the participants reporting findings in the same direction)], (3) indirectness (e.g. generalizability of the findings), (4) imprecision (downgraded when the total number of participants across studies is <300 for each outcome) and (5) other considerations, such as reporting bias. The quality of the evidence will be downgraded by one level when one of the factors described above is met (29, 34).

Two independent reviewers (ET, GT) will grade the quality of evidence. Single studies will be considered inconsistent and imprecise (i.e. sparse data) and providing ‘‘low quality evidence’’, which can be further downgraded to ‘‘very low quality evidence’’ if there are also limitations in design or indirectness. The following grading of quality of the evidence will be applied (33):

- High quality evidence: further research is very unlikely to change confidence in estimate of effect.
- Moderate quality evidence: further research is likely to have an important impact on confidence in estimate of effect and may change the estimate.

For peer review only

- Low quality evidence: further research is very likely to have an important impact on confidence in estimate of effect and is likely to change the estimate.
- Very low quality evidence: very little confidence in the effect estimate.
- No evidence: no RCTs were identified that addressed this outcome.

Patient and public involvement

The research question in this study forms part of a larger discussion within our patient and public involvement meetings as part of an existing programme of a multi-centre research programme that is focused on UQMD. Patients will not be involved in the data collection or analysis.

ETHICS AND DISSEMINATION

Ethics approval is not required for this systematic review. The study findings will be submitted to a relevant peer-reviewed journal for dissemination and presented at relevant conferences.

DISCUSSION

Thoracic spine manipuation (Tx-HVLAT) is reported to be one of the most often used treatment modality in multi-modal management strategies, not only for thoracic spinal pain but also for many other upper quandrant musculoskeletal disorders (UQMD).

Findings from this study may assist clinicians and researchers in formulating an individualised management plan for patients with UQMD. By assessing the effectiveness of Tx-HVLAT for different UQMD, clinicians will be better able to choose when to incorporate Tx-HVLAT as an effective treatment modality in evidence based multi-modal management strategies, instead of using a standardised ‘one size fits all’ approach.

Findings from this study will also serve a need both clinically and within the contemporary literature to inform further research (e.g. on efficacy, cost effectiveness, etc.). We also aim to compare and contrast this study’s findings with previously published systematic reviews (18, 19, 20, 21, 22, 24).

While results from relevant studies not published in English or Dutch or German might not be analyzed, we feel this will not impact the final outcome as it has been reported that exclusion of trials reported in a language other than English does not significantly affect the results of meta-analyses (35).

For peer review only

Contributors

MdG devised the initial focus of this systematic review with the help of all authors. ET and GT are PGR students, MdG is the lead supervisor, NH and DF are co-researchers. ET drafted the initial protocol manuscript with lead and co-supervisors providing guidance on methodological decisions and proposed analyses. All authors have contributed subject-specific expertise. All authors will contribute to data interpretation, conclusions and dissemination. All authors have read, contributed to and agreed to the final manuscript. DF and MdG are both guarantors of the study.

Funding

This study will be conducted as part of a Post Graduate Research project though the University of Birmingham, UK and as such received no specific grant from any funding agency in the public, commercial or not-for-profit sectors.

Competing interests

None declared.

Patient consent for publication

Not required.

Provenance and peer review

Not commissioned; externally peer reviewed.

Open access

This is an open access article distributed in accordance with the Creative Commons Attribution Non Commercial (CC BY-NC 4.0) license, which permits others to distribute, remix, adapt, build upon this work non-commercially, and license their derivative works on different terms, provided the original work is properly cited, appropriate credit is given, any changes made indicated, and the use is non-commercial.

Data availibility statement

Technical appendix, statistical code, and dataset available from the University of Birmingham eData Repository (https://edata.bham.ac.uk/).

Twitter

Erik Thoomes @Fysio_Experts
Deborah Falla @Deb_Falla
Nicola Heneghan @HeneghanNicola

For peer review only

REFERENCES

1. Huisstede BM, Bierma-Zeinstra SM, Koes BW, Verhaar JA. Incidence and prevalence of upper-extremity musculoskeletal disorders. A systematic appraisal of the literature. BMC musculoskeletal disorders. 2006;7:7.
2. Walker-Bone KE, Palmer KT, Reading I, Cooper C. Soft-tissue rheumatic disorders of the neck and upper limb: prevalence and risk factors. Semin Arthritis Rheum. 2003;33(3):185-203.
3. Executive H. Work-related Musculoskeletal Disorders (WRMSDs) Statistics in Great Britain. 2017. 2016.
4. Bier JD, Scholten-Peeters WGM, Staal JB, Pool J, van Tulder MW, Beekman E, et al. Clinical Practice Guideline for Physical Therapy Assessment and Treatment in Patients With Nonspecific Neck Pain. Phys Ther. 2018;98(3):162-71.
5. Blanpied PR, Gross AR, Elliott JM, Devaney LL, Clewley D, Walton DM, et al. Neck Pain: Revision 2017. The Journal of orthopaedic and sports physical therapy. 2017;47(7):A1-a83.
6. Lafrance S, Charron M, Roy JS, Dyer JO, Frémont P, Dionne CE, et al. Diagnosing, Managing, and Supporting Return to Work of Adults With Rotator Cuff Disorders: A Clinical Practice Guideline. The Journal of orthopaedic and sports physical therapy. 2022;52(10):647-64.
7. Lapner P, Alfonso A, Herbert-Davies J, Pollock JW, Marsh J, King G. Position statement: nonoperative management of lateral epicondylitis in adults. Can J Surg. 2022;65(5):E625-e9.
8. Yu H, Côté P, Wong JJ, Shearer HM, Mior S, Cancelliere C, et al. Noninvasive management of soft tissue disorders of the shoulder: A clinical practice guideline from the Ontario Protocol for Traffic Injury Management (OPTIMa) collaboration. European journal of pain (London, England). 2021;25(8):1644-67.
9. Hegmann KT, Hoffman HE, Belcourt RM, Byrne K, Glass L, Melhorn JM, et al. ACOEM practice guidelines: elbow disorders. J Occup Environ Med. 2013;55(11):1365-74.
10. Rusthon A ea. The Standards Document, Glossary of Terms: IFOMPT; 2012 [Available from: http://www.ifompt.com/Standards/SC+Glossary.html.
11. Heneghan NR, Puentedura EJ, Arranz I, Rushton A. Thoracic thrust joint manipulation: An international survey of current practice and knowledge in IFOMPT member countries. Musculoskeletal science & practice. 2020;50:102251.
12. Triano JJ, Rogers CM, Combs S, Potts D, Sorrels K. Quantitative feedback versus standard training for cervical and thoracic manipulation. Journal of manipulative and physiological therapeutics. 2003;26(3):131-8.
13. Adams G, Sim J. A survey of UK manual therapists' practice of and attitudes towards manipulation and its complications. Physiotherapy research international : the journal for researchers and clinicians in physical therapy. 1998;3(3):206-27.
14. Puentedura EJ, Slaughter R, Reilly S, Ventura E, Young D. Thrust joint manipulation utilization by U.S. physical therapists. The Journal of manual & manipulative therapy. 2017;25(2):74-82.
15. Heneghan NR, Rushton A. Understanding why the thoracic region is the 'Cinderella' region of the spine. Man Ther. 2016;21:274-6.
16. Côté P, Wong JJ, Sutton D, Shearer HM, Mior S, Randhawa K, et al. Management of neck pain and associated disorders: A clinical practice guideline from the Ontario Protocol for Traffic Injury Management (OPTIMa) Collaboration. European Spine Journal. 2016;25(7):2000-22.
17. Thoomes E, Graaf MT, Cleland JA, Gallina A, Falla D. Timing of Evidence-Based Nonsurgical Interventions as Part of Multimodal Treatment Guidelines for the Management of Cervical Radiculopathy: A Delphi Study. Phys Ther. 2022.
18. Cross KM, Kuenze C, Grindstaff TL, Hertel J. Thoracic spine thrust manipulation improves pain, range of motion, and self-reported function in patients with mechanical neck

For peer review only

pain: a systematic review. The Journal of orthopaedic and sports physical therapy. 2011;41(9):633-42.
19. Huisman PA, Speksnijder CM, de Wijer A. The effect of thoracic spine manipulation on pain and disability in patients with non-specific neck pain: a systematic review. Disabil Rehabil. 2013;35(20):1677-85.
20. Masaracchio M, Kirker K, States R, Hanney WJ, Liu X, Kolber M. Thoracic spine manipulation for the management of mechanical neck pain: A systematic review and meta-analysis. PloS one. 2019;14(2):e0211877.
21. Tsegay GS, Gebregergs GB, Weleslassie GG, Hailemariam TT. Effectiveness of Thoracic Spine Manipulation on the Management of Neck Pain: A Systematic Review and Meta-Analysis of Randomized Control Trials. Journal of pain research. 2023;16:597-609.
22. Bini P, Hohenschurz-Schmidt D, Masullo V, Pitt D, Draper-Rodi J. The effectiveness of manual and exercise therapy on headache intensity and frequency among patients with cervicogenic headache: a systematic review and meta-analysis. Chiropractic & manual therapies. 2022;30(1):49.
23. Strunce JB, Walker MJ, Boyles RE, Young BA. The immediate effects of thoracic spine and rib manipulation on subjects with primary complaints of shoulder pain. The Journal of manual & manipulative therapy. 2009;17(4):230-6.
24. Sanzo P, Yeung E, Levesque L, Maheu E, Woodard T, Michels S, et al. Is thoracic manipulation effective in managing shoulder dysfunction–A systematic review. Man Ther. 2016;100(25):e154.
25. Zunke P, Auffarth A, Hitzl W, Moursy M. The effect of manual therapy to the thoracic spine on pain-free grip and sympathetic activity in patients with lateral epicondylalgia humeri. A randomized, sample sized planned, placebo-controlled, patient-blinded monocentric trial. BMC musculoskeletal disorders. 2020;21(1):186.
26. Page MJ, McKenzie JE, Bossuyt PM, Boutron I, Hoffmann TC, Mulrow CD, et al. The PRISMA 2020 statement: an updated guideline for reporting systematic reviews. BMJ (Clinical research ed). 2021;372:n71.
27. Higgins JP, Thomas J, Chandler J, Cumpston M, Li T, Page MJ, et al. Cochrane handbook for systematic reviews of interventions: John Wiley & Sons; 2019.
28. Sterne JAC, Savović J, Page MJ, Elbers RG, Blencowe NS, Boutron I, et al. RoB 2: a revised tool for assessing risk of bias in randomised trials. BMJ (Clinical research ed). 2019;366:l4898.
29. Atkins D, Best D, Briss PA, Eccles M, Falck-Ytter Y, Flottorp S, et al. Grading quality of evidence and strength of recommendations. BMJ (Clinical research ed). 2004;328(7454):1490.
30. Higgins JP, Thomas J, Chandler J, Cumpston M, Li T, Page MJ, et al. Cochrane handbook for systematic reviews of interventions; v 6.3: John Wiley & Sons; 2022.
31. Dettori JR, Norvell DC, Chapman JR. Fixed-Effect vs Random-Effects Models for Meta-Analysis: 3 Points to Consider. Global spine journal. 2022;12(7):1624-6.
32. Landis JR, Koch GG. The measurement of observer agreement for categorical data. Biometrics. 1977;33(1):159-74.
33. GRADE-Pro. GRADE handbook for grading quality of evidence and strength of recommendations. Updated. Schünemann H BJ, Guyatt G, Oxman A, editors, editor: The GRADE Working Group; 2013.
34. Brozek JL, Akl EA, Alonso-Coello P, Lang D, Jaeschke R, Williams JW, et al. Grading quality of evidence and strength of recommendations in clinical practice guidelines. Part 1 of 3. An overview of the GRADE approach and grading quality of evidence about interventions. Allergy. 2009;64(5):669-77.
35. Moher D, Pham B, Lawson ML, Klassen TP. The inclusion of reports of randomised trials published in languages other than English in systematic reviews. Health technology assessment (Winchester, England). 2003;7(41):1-90.

For peer review only

PIO: What is the effectiveness of a thoracic high velocity low amplitude thrust (HVLAT) manipulation in patients with musculoskeletal disorders in the upper quadrant, a systematic review.

OVID search of Embase and Medline

Musculoskeletal adj2 (pain OR disorder* OR disease* OR symptom* OR problem*) OR Neck adj2 (pain OR disorder* OR disease* OR symptom* OR problem*) OR Headache adj2 (disorder* OR tension type OR cervicogenic OR migraine) OR (Cervical radiculopathy) OR (radicular adj2 (syndrome* OR pain)) OR Cervical adj2 (nerve root OR disc OR (disc disease) OR impingement) OR Shoulder adj2 (pain OR disorder* OR disease* OR symptom* OR problem* OR imping*) OR (adhesive capsulitis) OR (frozen shoulder) OR Elbow adj2 (pain OR disorder* OR disease* OR symptom* OR problem* OR tennis) OR epicondylalgia OR epicondylitis OR Temporomandibular adj2 (joint OR pain OR disorder* OR disease* OR symptom* OR problem*) OR Upper extremity adj2 (joint OR pain OR disorder* OR disease* OR symptom* OR problem*) OR Upper quadrant adj2 (joint OR pain OR disorder* OR disease* OR symptom* OR problem*)

AND

Thoracic OR (Thoracic adj2 spine) OR Thora*

AND

Spinal manipulation OR Manipulation OR manipulat* OR adjustment OR Chiroprac* OR (high adj2 (velocity OR low OR amplitude OR thrust)) OR treat* OR therapy OR intervention OR SMT OR thrust OR mobilis* OR mobiliz* OR mobilization OR mobilization OR manual therapy OR osteopat* OR physical therapy OR physio* OR rehab* OR management OR HVLA*

AND

Pain OR (numerical rating scale) OR NRS OR (numerical pain rating scale) OR NPRS OR Disability OR disable* OR (return to work) OR effectiveness OR effect* OR patient adj2 (reported OR outcome OR measur*) OR (recovery of function) OR activit* adj2 (daily OR living OR life) OR Physical adj2 (examination OR test) OR Range adj2 motion OR Muscle adj2 strength OR Psychosocial* OR (Neck disability index) OR NDI OR (Bournemouth adj2 neck) OR (patient specific functional scale) OR PSFS OR (shoulder pain and disability index) OR SPADI
AND

For peer review only

Web of Science:

Musculoskeletal NEAR/1 (pain OR disorder* OR disease* OR symptom* OR problem*) OR Neck NEAR/1 (pain OR disorder* OR disease* OR symptom* OR problem*) OR Headache NEAR/1 (disorder* OR “tension type” OR cervicogenic OR migraine) OR (Cervical radiculopathy) OR (radicular NEAR/1 (syndrome* OR pain)) OR Cervical NEAR/1 ((“nerve root” OR disc OR “disc disease”) OR impingement) OR Shoulder NEAR/1 (pain OR disorder* OR disease* OR symptom* OR problem* OR imping*) OR “adhesive capsulitis” OR “frozen shoulder” OR Elbow NEAR/1 (pain OR disorder* OR disease* OR symptom* OR problem* OR tennis) OR epicondylalgia OR epicondylitis OR Temporomandibular NEAR/1 (joint OR pain OR disorder* OR disease* OR symptom* OR problem*) OR Upper extremity NEAR/1 (joint OR pain OR disorder* OR disease* OR symptom* OR problem*) OR Upper quadrant NEAR/2 (joint OR pain OR disorder* OR disease* OR symptom* OR problem*)

AND

Thoracic OR (Thoracic NEAR/2 spine OR spinal) OR Thoracal

AND

Spinal manipulation OR Manipulation OR manipulat* OR adjustment OR Chiroprac* OR (high NEAR/1 (velocity OR low OR amplitude OR thrust)) OR treat* OR therapy OR intervention OR smt OR thrust OR mobilis* OR mobiliz* OR mobilization OR mobilization OR manual therapy OR osteopat* OR physical therapy OR physio* OR rehab* OR management OR HVLA*

AND

Pain OR measure* OR (numerical rating scale) OR NRS OR (numerical pain rating scale) OR NPRS OR Disability OR disable* OR (return to work) OR patient NEAR/1 (reported OR outcome OR measur*) OR (recovery of function) OR activit* NEAR/1 (daily OR living OR life) OR Physical NEAR/1 (examination OR test) OR Range NEAR/1 motion OR Muscle NEAR/1 strength OR Psychosocial* OR (Neck disability index) OR NDI OR (Bournemouth NEAR/1 neck) OR (patient specific functional scale) OR PSFS OR (shoulder pain and disability index) OR SPADI

AND

randomised OR randomized OR random* OR (Clinical trial) OR RCT

SCOPUS; also used for CINAHL

Musculoskeletal W/2 pain OR Musculoskeletal W/2 disorder* OR Musculoskeletal W/2 disease* OR Musculoskeletal W/2 symptom* OR Musculoskeletal W/2 problem* OR Neck W/2 pain OR Neck W/2 disorder* OR Neck W/2 disease* OR Neck W/2 symptom* OR Neck W/2 problem* OR Headache W/2 disorder* OR Headache W/2 “tension type” OR Headache W/2 cervicogenic OR Headache W/2 migraine OR “Cervical radiculopathy” OR radicular W/2 syndrome* OR radicular W/2 pain OR radicular W/2 Cervical W/2 “nerve root” OR Cervical W/2 disc OR Cervical W/2 “disc disease” OR Cervical W/2 impingement OR Shoulder W/2 pain OR Shoulder W/2 disorder* OR Shoulder W/2 disease* OR Shoulder W/2 symptom* OR Shoulder W/2 problem* OR Shoulder W/2 imping* OR “adhesive capsulitis” OR “frozen shoulder” OR Elbow W/2

For peer review only

pain OR Elbow W/2 disorder* OR Elbow W/2 disease* OR Elbow W/2 symptom* OR Elbow W/2 problem* OR Elbow W/2 tennis) OR epicondylalgia OR epicondylitis OR Temporomandibular W/2 joint OR Temporomandibular W/2 pain OR Temporomandibular W/2 disorder* OR Temporomandibular W/2 disease* OR Temporomandibular W/2 symptom* OR Temporomandibular W/2 problem* OR "Upper extremity" W/2 joint OR "Upper extremity" pain OR "Upper extremity" disorder* OR "Upper extremity" disease* OR "Upper extremity" symptom* OR "Upper extremity" problem* OR "Upper quadrant" W/2 joint OR "Upper quadrant" W/2 pain OR "Upper quadrant" W/2 disorder* OR "Upper quadrant" W/2 disease* OR "Upper quadrant" W/2 symptom* OR "Upper quadrant" W/2 problem*

AND

Thoracic OR Thoracic W/2 spine OR Thoracic W/2 spinal OR Thora*

AND

"Spinal manipulation" OR Manipulation OR manipulat* OR adjustment OR Chiroprac* OR high W/2 velocity OR high W/2 low OR high W/2 amplitude OR high W/2 thrust OR treat* OR therapy OR intervention OR SMT OR "spinal manipul* therapy" OR thrust OR mobilis* OR mobiliz* OR mobilization OR mobilization OR manual therapy OR osteopat* OR "physical therapy" OR physio* OR rehab* OR management OR HVLA* OR "manipulative technique*"

AND

Pain OR measure* OR "numerical rating scale" OR NRS OR "numerical pain rating scale" OR NPRS OR Disability OR disable* OR "return to work" OR effectiveness OR effect* OR patient W/4 reported OR patient W/4 outcome OR patient W/4 measur* OR "recovery of function" OR activit* W/2 daily OR activit* W/2 living OR activit* W/2 life OR Physical W/2 examination OR Physical W/2 test OR Range W/2 motion OR Muscle W/2 strength OR Psychosocial* OR "neck disability index" OR NDI OR Bournemouth W/2 neck OR Bournemouth W/2 question* OR "patient specific functional scale" OR PSFS OR "shoulder pain and disability index" OR spadi

AND

randomised OR randomized OR random* OR "Clinical trial" OR RCT

Index to Chiropractic Literature

'Thoracic manipulation' AND random*

PEDro database

Thorac* AND manip*

For peer review only
